# Heterogeneity in the distribution of 159 drug-response related SNPs in world populations and their genetic relatedness

**DOI:** 10.1371/journal.pone.0228000

**Published:** 2020-01-23

**Authors:** Tamim Ahsan, Nusrat Jahan Urmi, Abu Ashfaqur Sajib

**Affiliations:** 1 Department of Genetic Engineering & Biotechnology, Bangabandhu Sheikh Mujibur Rahman Maritime University, Dhaka, Bangladesh; 2 Department of Medicine, BIRDEM General Hospital, Dhaka, Bangladesh; 3 Department of Genetic Engineering & Biotechnology, University of Dhaka, Dhaka, Bangladesh; Ohio State University Wexner Medical Center, UNITED STATES

## Abstract

Interethnic variability in drug response arises from genetic differences associated with drug metabolism, action and transport. These genetic variations can affect drug efficacy as well as cause adverse drug reactions (ADRs). We retrieved drug-response related single nucleotide polymorphism (SNP) associated data from databases and analyzed to elucidate population specific distribution of 159 drug-response related SNPs in twenty six populations belonging to five super-populations (African, Admixed Americans, East Asian, European and South Asian). Significant interpopulation differences exist in the minor (variant) allele frequencies (MAFs), linkage disequilibrium (LD) and haplotype distributions among these populations. 65 of the drug-response related alleles, which are considered as minor (variant) in global population, are present as the major alleles (frequency ≥0.5) in at least one or more populations. Populations that belong to the same super-population have similar distribution pattern for majority of the variant alleles. These drug response related variant allele frequencies and their pairwise LD measure (r^2^) can clearly distinguish the populations in a way that correspond to the known evolutionary history of human and current geographic distributions, while D' cannot. The data presented here may aid in identifying drugs that are more appropriate and/or require pharmacogenetic testing in these populations. Our findings emphasize on the importance of distinct, ethnicity-specific clinical guidelines, especially for the African populations, to avoid ADRs and ensure effective drug treatment.

## Introduction

Pharmacogenomics studies interindividual variability in drug response, which is mainly caused by particular genetic variants associated with drug absorption, distribution, metabolism and elimination (ADME) [1, 2]. Differences in drug response can also be caused by variants in leukocyte antigen genes and drug targets [3]. These variants can modulate efficacy of drugs as well as result in ADRs, which are major causes of hospitalizations and mortalities in both adults and children [4–7]. Such adverse reactions not only exacerbate the patients’ illness, but also cause economic losses [8]. However, ADRs may be avoided in many cases if the genotypes of the patients at the drug-response related loci are known. For example, genotype-guided warfarin dosing was shown to significantly reduce warfarin-related internal bleeding and thromboembolism [9].

Except for a small fraction of the total genetic variants, the majority (genetic variants with minor allele frequencies > 0.05) are commonly shared across populations [10]. But this tiny fraction of the total genetic variants distinguish between metabolic phenotypes of the continental populations [11]. Besides, there is evidence of interethnic and intraethnic differences in the distribution of drug-response associated genetic variants and, as a consequence, variability in drug responses [12–14]. For example, rosuvastatin is commonly prescribed to prevent cardiovascular complications and treat abnormal lipid levels in the blood. Although its high efficacy and safety profile as a drug to tackle dyslipidemia are well-known, multiple studies have reported dose-dependent adverse effects of prolonged statin therapy [15–17]. Ethnic differences exist in the pharmacokinetics of rosuvastatin. The average systemic exposure to this drug among the individuals of Chinese ethnicity is 2.3-fold greater than the Caucasians, whereas Malays and Asian Indians have intermediate values [18].

Due to interpopulation genetic variations, drugs as well as markers used for pharmacogenotyping in one population may not be appropriate for another population. For example, HLA-b*58:01 allele is associated with allopurinol-induced severe cutaneous adverse reactions and rs9263726 can be used as a surrogate biomarker for the Japanese, but not the Australian and the Han Chinese populations [19, 20]. Population-based differences in the outcomes of anticancer treatments have also been reported. For example, discrepant responses to 5-Fluorouracil (5-FU) among different ethnicities of the South Asian population were attributed to genetic variations in the DPYD gene [21]. Analysis of population specific genetic structure, therefore, has many applications in medical and population genetic research as well as ensuring drug efficacy and development of pharmacogenetic tests [8, 22, 23].

Many aspects of the population history are reflected in genetic information [23]. SNPs and their allelic distribution provide important information about population structure, evolution and migration [24–29]. There are population-specific differences in the extent and pattern of linkage disequilibrium (LD) among genetic variants [11]. Levels and patterns of LD depend on a number of demographic factors such as population size and structure, population growth, admixture, migration and locus-specific factors such as mutation, selection, recombination, gene conversion and genetic drift [30, 31]. The application and transferability of surrogate biomarkers and/or tagSNPs from a particular genome wide association study (GWAS) depends on the genetic relatedness between the studied populations [32–35]. Hence, it is important to know population-specific LD patterns among different genetic variants before widely implementing results of GWAS.

Since allelic distribution and linkage disequilibrium (LD) of SNPs vary among populations, frequencies of different SNP alleles associated with drug response and patterns of LD should be analyzed separately for different populations. Here, we present the variant allele distribution, pairwise LD and haplotypes frequencies of 159 drug-response associated SNPs in five super-populations (African, Admixed Americans, East Asian, European and South Asian) and twenty six individual populations belonging to these super-populations.

## Materials and methods

### List of SNPs associated with drug response

The dbSNP database (https://www.ncbi.nlm.nih.gov/snp) at the National Center for Biotechnology Information (NCBI) was searched using the keyword ‘drug-response’. After filtering out the duplicates and insertion/deletion (indel) polymorphisms, 159 SNPs were selected for further analysis. Drugs related to these SNPs along with their applications were searched in ClinVar archive at NCBI (https://www.ncbi.nlm.nih.gov/clinvar) [36] and the PharmGKB (https://www.pharmgkb.org/) [37].

### Allele frequency and pairwise LD calculation

We used the LDhap module at LDlink (https://ldlink.nci.nih.gov/) [38] to retrieve the population-specific allele and haplotype frequencies from the phase 3 (version 5) sequence data of the 1000 Genomes Project [39] for five super-populations (African, Admixed Americans, East Asian, European and South Asian) and twenty six individual populations belonging to these super-populations (Listed in Table 1). LDlink is a suite of web-based bioinformatics modules that provides an easy and user-friendly interface to investigate SNPs, LD and haplotypes in populations included in the 1000 Genomes Project [38]. The Reference SNP (rs) numbers of the SNPs were used as inputs. We used the LDmatrix module at LDlink to calculate the pairwise LD among the SNPs in different super- and sub-populations. SNPs that are located on the same chromosome were inputted together. SNP pairs that maintain a strong LD (r^2^≥ 0.8) were selected and compiled in a non-redundant list.

**Table 1:** List of drug-response related SNPs with MAF ≥0.5.

SNP ID	Global major allele	Global minor allele	Associated drug	Drug used for	Population^#^																															
					ALL	AFR								AMR					EAS						EUR						SAS					
					All pops	AFR	YRI	LWK	GWD	MSL	ESN	ASW	ACB	AMR	MXL	PUR	CLM	PEL	EAS	CHB	JPT	CHS	CDX	KHV	EUR	CEU	TSI	FIN	GBR	IBS	SAS	GIH	PJL	BEB	STU	ITU
rs1801133	G	A	Cyclophosphamide, carboplatin, methotrexate	Precursor cell lymphoblastic leukemia-lymphoma, carcinoma, non-small-cell lung cancer, neoplasms	0.25												0.54																			
rs2297480	T	G	Bisphosphonates	Postmenopausal osteoporosis	0.37														0.69	0.76	0.74	0.75	0.66	0.54												
rs1801274	A	G	Trastuzumab	Breast neoplasms	0.44	0.53	0.52			0.61	0.60				0.51										0.51			0.54	0.61	0.53						
rs1051740	T	C	Carbamazepine	Epilepsy	0.31																			0.54												
rs1056836	G	C	-	Congenital glaucoma	0.39	0.82	0.88	0.79	0.82	0.87	0.84	0.70	0.79																							
rs6166	T	C	Follice-stimulating hormone	Ovarian hyperstimulation syndrome, ovarian response to FSH stimulation, ovarian dysgenesis	0.41																							0.50				0.51				
rs6165	C	T	Follice-stimulating hormone	Ovarian hyper-stimulation syndrome, ovarian response to FSH stimulation, ovarian dysgenesis	0.49									0.58	0.67		0.56	0.66	0.66	0.67	0.64	0.67	0.67	0.65	0.55	0.59	0.53	0.50	0.56	0.57	0.54		0.52	0.63	0.55	0.54
rs3812718	C	T	Carbamazepine, phenytoin, anti-epileptics	Epilepsy	0.49												0.56		0.60		0.51	0.62	0.68	0.70	0.54	0.53	0.53	0.57	0.61		0.58	0.58	0.58	0.60	0.59	0.55
rs2952768	T	C	Fentanyl, morphine, opioids	Pain	0.39													0.54													0.51			0.51	0.52	0.57
rs4673993	T	C	Methotrexate	Rheumatoid arthritis	0.29																															0.56
rs887829	C	T	Atazanavir	Human immunodeficiency virus infection	0.35		0.52	0.53																												
rs4961	G	T	Furosemide, spironolactone	Liver cirrhosis	0.21															0.54	0.56															
rs145489027	G	A	ACE inhibitor		0.30																		0.55	0.51												
rs1902023	C	A	Oxazepam, lorazepam	Anxiety	0.45																				0.51	0.55	0.51	0.52	0.54		0.54	0.55			0.64	0.55
rs4693075	C	G	Atorvastatin, HMG CoA reductase inhibitors, rosuvastatin	Muscular diseases	0.34							0.53						0.54																		
rs4444903	G	A	Cetuximab	Colorectal neoplasms, rectal neoplasms	0.40																				0.61	0.60	0.62	0.63	0.59	0.61		0.51	0.52			
rs1801394	A	G	Methotrexate	Burkitt lymphoma, T-cell precursor cell lymphoblastic leukemia-lymphoma, lymphoma, toxic liver disease, gastrointestinal stroma tumor, disorders of intracellular cobalamin metabolism	0.36																				0.52	0.56		0.62	0.57		0.52		0.51	0.54	0.51	0.59
rs6295	G	C	Paroxetine	Depressive disorder, panic disorder 1, major mood disorders	0.45	0.57	0.59	0.61	0.56	0.55	0.54	0.57	0.57		0.58		0.52								0.54	0.51	0.59	0.61		0.53						
rs17244841	A	T	HMG CoA reductase inhibitors, pravastatin, simvastatin	Coronary artery disease, hyperlipidemias, coronary disease, myocardial infarction, hypercholesterolemia	0.04																															
rs1042713	G	A	Salbutamol, salmeterol	Asthma	0.48	0.52	0.53		0.53	0.52	0.52	0.55	0.51				0.52		0.55	0.55		0.61	0.58	0.57												
rs20455	G	A	Pravastatin, atorvastatin	Coronary disease, myocardial infarction, hyperlipidemias	0.46									0.67	0.68	0.59	0.62	0.79		0.52	0.51				0.64	0.63	0.65	0.66	0.66	0.59	0.54	0.54	0.55	0.56	0.52	0.52
rs1799971	A	G	Ethanol, alfentanil, fentanyl, heroin, morphine, naltrexone, opioids, tramadol, buprenorphine, drugs used in opioid dependence	Alcohol dependence, heroin dependence, opioid-related disorders, pain	0.22																															
rs4880	A	G	Cyclophosphamide	Breast neoplasms	0.41									0.58	0.65	0.54		0.68											0.52		0.51	0.51			0.58	
rs37973	A	G	Glucocorticoids		0.40													0.51				0.51		0.52							0.51	0.53		0.59		0.52
rs7793837	A	T	Salbutamol, selective beta-2-adrenoreceptor agonists	Drug reported used for: Asthma, Drug reported used for: Asthma	0.42	0.86	0.91	0.84	0.87	0.86	0.91	0.76	0.81																							
rs1045642	G	A	Nevirapine, methotrexate, fentanyl, methadone, morphine, opioids, oxycodone, tramadol, digoxin	HIV Infections, toxic epidermal necrolysis, Burkitt lymphoma, precursor cell lymphoblastic leukemia-lymphoma, lymphoma, toxic liver disease, pain, heroin dependence	0.40																				0.52	0.57		0.58	0.53		0.58	0.57	0.51	0.61	0.59	0.59
rs776746	C	T	Cyclosporine, tacrolimus, sirolimus	Organ transplantation, kidney transplantation, liver transplantation	0.38	0.82	0.83	0.88	0.77	0.88	0.89	0.69	0.75																							
rs2740574	T	C	Condition: tacrolimus response—Dosage	Drug reported used for: Organ Transplantation	0.23	0.77	0.76	0.83	0.79	0.84	0.77	0.67	0.66																							
rs10246939	C	T	Phenylthio-carbamide tasting		0.48	0.52	0.54	0.51	0.58	0.51	0.53		0.54												0.54	0.57		0.62	0.58	0.50	0.64	0.58	0.59	0.67	0.70	0.66
rs1726866	G	A	Phenylthio-carbamide tasting		0.43																				0.54	0.57		0.62	0.58		0.64	0.58	0.59	0.67	0.70	0.66
rs713598	G	C	Phenylthio-carbamide tasting		0.50	0.53	0.54	0.50	0.58	0.51	0.52		0.54												0.58	0.62		0.63	0.61	0.55	0.66	0.60	0.62	0.68	0.70	0.68
rs6977820	C	T	Antipsychotics	Metabolic syndrome X, schizophrenia, hyper-prolactinemia, tardive dyskinesia, weight gain, mental disorders	0.42	0.83	0.88	0.83	0.84	0.85	0.90	0.68	0.74																							
rs1041983	C	T	Slow acetylator due to N-acetyl transferase enzyme variant, ethambutol, isoniazid, pyrazinamide, rifampin	Tuberculosis	0.40					0.50	0.53													0.54											0.50	
rs6988229	C	T	Salbutamol	Asthma	0.24	0.60	0.62	0.57	0.62	0.59	0.59	0.57	0.60																							
rs1695	A	G	Platinum compounds, fluorouracil, oxaliplatin, cyclophosphamide, epirubicin, cisplatin	Neoplasms, ovarian neoplasms, colorectal neoplasms, breast neoplasms, osteosarcoma, urinary bladder neoplasms, medulloblastoma, brain neoplasms	0.35			0.51	0.54		0.54				0.56			0.67																		
rs716274	A	G	Platinum compounds	Neoplasms, ovarian neoplasms	0.44				0.50			0.54				0.51									0.59	0.58	0.63	0.59	0.57	0.60			0.51		0.50	
rs11212617	C	A	Metformin	Diabetes mellitus	0.47									0.64	0.59	0.61	0.66	0.69							0.62	0.55	0.69	0.58	0.59	0.66	0.63	0.64	0.70	0.61	0.56	0.62
rs1954787	C	T	Antidepressants	Depression, depressive disorder, major depressive disorder, mood disorders	0.50	0.90	0.93	0.86	0.95	0.94	0.90	0.83	0.83															0.52								
rs5443	C	T	Sildenafil	Erectile dysfunction	0.49	0.82	0.85	0.75	0.82	0.89	0.81	0.78	0.83						0.50			0.50	0.58	0.55												
rs11045879	T	C	Methotrexate	Burkitt lymphoma, precursor cell lymphoblastic leukemia-lymphoma, toxic liver disease	0.22																		0.54													
rs7997012	G	A	Antidepressants, citalopram, selective serotonin reuptake inhibitors	Depression, depressive disorder, major depressive disorder	0.27																							0.53				0.52				
rs1719247	T	C	HMG CoA reductase inhibitors, simvastatin	Muscular diseases, myopathy	0.41											0.60	0.53								0.73	0.80	0.71	0.67	0.74	0.73	0.55	0.57	0.61	0.51	0.51	0.57
rs1346268	T	C	HMG CoA reductase inhibitors, simvastatin	Muscular diseases, myopathy	0.45									0.52	0.61			0.79	0.81	0.85	0.88	0.82	0.80	0.68												
rs578776	A	G	Nicotine	Tobacco use disorder	0.45												0.53								0.72	0.75	0.69	0.70	0.71	0.74	0.51	0.52	0.53		0.57	0.53
rs7294	C	T	Warfarin, acenocoumarol, phenprocoumon, vitamin K-dependent clotting factors	Heart diseases, atrial fibrillation, arteriosclerosis, hemorrhage, intracranial hemorrhages, myocardial infarction, peripheral vascular diseases, thromboembolism, venous thromboembolism, pulmonary embolism, stroke	0.42		0.51											0.57													0.76	0.67	0.69	0.79	0.82	0.83
rs2359612	G	A	Warfarin	Heart diseases, atrial fibrillation, arteriosclerosis, hemorrhage, intracranial hemorrhages, myocardial infarction, peripheral vascular diseases, thromboembolism, venous thromboembolism, pulmonary embolism, stroke	0.39														0.89	0.96	0.90	0.89	0.82	0.84												
rs8050894	C	G	Warfarin	Heart diseases, atrial fibrillation, arteriosclerosis, hemorrhage, intracranial hemorrhages, myocardial infarction, peripheral vascular diseases, thromboembolism, venous thromboembolism, pulmonary embolism, stroke	0.42										0.51				0.89	0.96	0.90	0.89	0.82	0.84			0.50									
rs9934438	G	A	Warfarin, acenocoumarol, phenprocoumon, vitamin K-dependent clotting factors	Heart diseases, atrial fibrillation, arteriosclerosis, hemorrhage, intracranial hemorrhages, myocardial infarction, peripheral vascular diseases, thromboembolism, venous thromboembolism, pulmonary embolism, stroke	0.36														0.89	0.96	0.90	0.89	0.82	0.84												
rs9923231	C	T	Warfarin, acenocoumarol, phenprocoumon	Heart diseases, atrial fibrillation, arteriosclerosis, hemorrhage, intracranial hemorrhages, myocardial infarction, peripheral vascular diseases, thromboembolism, venous thromboembolism, pulmonary embolism, stroke	0.36														0.89	0.96	0.90	0.89	0.82	0.84												
rs7196161	A	G	Warfarin	Heart diseases, atrial fibrillation, arteriosclerosis, hemorrhage, intracranial hemorrhages, myocardial infarction, peripheral vascular diseases, thromboembolism, venous thromboembolism, pulmonary embolism, stroke	0.47					0.61	0.51								0.89	0.96	0.90	0.89	0.82	0.84												
rs1532624	C	A	HMG CoA reductase inhibitors	Coronary artery disease, hyperlipidemias	0.31																											0.54				0.52
rs2232228	A	G	Anthracyclines and related substances	Heart failure, cardiomyopathies, neoplasms	0.34										0.50				0.52	0.54		0.50	0.57	0.51												
rs1042522	C	G	Antineoplastic agents, cisplatin, cyclophosphamide, fluorouracil, paclitaxel	Breast neoplasms, neoplasms, neutropenia, ovarian neoplasms, stomach neoplasms, non-small-cell lung carcinoma, colorectal neoplasms, esophageal neoplasms, mesothelioma, pancreatic neoplasms, uterine cervical neoplasms	0.46	0.67	0.64	0.75	0.71	0.61	0.68	0.60	0.66																				0.52		0.54	0.54
rs4149601	G	A	Diuretics, hydrochlorothiazide	Hypertension, essential hypertension	0.28			0.50																												
rs17782313	T	C	Antipsychotics	Metabolic syndrome X, schizophrenia, hyper-prolactinemia, tardive dyskinesia, weight gain, mental Disorders	0.24																															
rs489693	C	A	Amisulpride, aripiprazole, clozapine, haloperidol, olanzapine, paliperidone, quetiapine, risperidone, ziprasidone	Autism spectrum disorder, schizoaffective disorder, schizophrenia. Metabolic syndrome X, hyper-prolactinemia, tardive dyskinesia, weight gain	0.35	0.50	0.53		0.53	0.58			0.50																							
rs12979860	C	T	Peginterferon alfa-2a, peginterferon alfa-2b, and ribavirin, telaprevir, boceprevir	Hepatitis C, HIV infection, chronic Hepatitis C infection	0.36	0.67	0.68	0.52	0.73	0.65	0.71	0.68	0.71																							
rs3212986	C	A	Cisplatin, platinum, platinum compounds	Neoplasms, osteosarcoma, urinary bladder neoplasms, ovarian neoplasms, medulloblastoma, brain neoplasms	0.30										0.50																					
rs11615	G	A	Carboplatin, cisplatin, oxaliplatin, platinum compounds	Non-small-cell lung carcinoma, colorectal neoplasms, esophageal neoplasms, mesothelioma, ovarian neoplasms, pancreatic neoplasms, breast neoplasms, stomach neoplasms, cervical neoplasms	0.33																				0.62	0.64	0.54	0.63	0.68	0.63		0.52	0.51			
rs1056892	G	A	Anthracyclines and related substances	Heart failure, cardiomyopathies, neoplasms	0.43	0.51	0.51		0.53	0.52	0.57		0.53																		0.53	0.54		0.59	0.53	0.52
rs4680	G	A	Nicotine	Tobacco use disorder	0.37																				0.50			0.59	0.53				0.52			
rs2298383	C	T	Caffeine		0.40									0.52	0.65	0.50	0.51			0.52		0.56			0.59	0.59	0.58	0.57	0.63	0.57						
rs1135840	G	C	Debrisoquine	Ultra-rapid metabolism of debrisoquine	0.40									0.52	0.60		0.51	0.61			0.50							0.53					0.57	0.50		
rs16947	G	A	Debrisoquine	Ultra-rapid metabolism of debrisoquine	0.36	0.55	0.56	0.65	0.54	0.61	0.57																									
rs1065852	G	A	Debrisoquine	Ultra-rapid metabolism of debrisoquine	0.24														0.57	0.60		0.61	0.63	0.66												

^**#**^ All populations (**ALL**); African super-population **(AFR)- ((**Yoruba in Ibadan, Nigeria **(YRI);** Luhya in Webuye, Kenya **(LWK);** Gambian in Western Divisions in the Gambia **(GWD);** Mende in Sierra Leone **(MSL);** Esan in Nigeria **(ESN);** Americans of African Ancestry in SW USA **(ASW);** African Caribbeans in Barbados **(ACB));** Ad Mixed Americans (**AMR)- ((**Mexican Ancestry from Los Angeles USA **(MXL);** Puerto Ricans from Puerto Rico **(PUR);** Colombians from Medellin, Colombia **(CLM);** Peruvians from Lima, Peru **(PEL));** East Asian (**EAS**)- ((Han Chinese in Beijing, China **(CHB);** Japanese in Tokyo, Japan **(JPT);** Southern Han Chinese **(CHS);** Chinese Dai in Xishuangbanna, China **(CDX);** Kinh in Ho Chi Minh City, Vietnam **(KHV)**); European (**EUR)- ((**Utah Residents (CEPH) with Northern and Western European Ancestry **(CEU);** Toscani in Italia **(TSI);** Finnish in Finland **(FIN);** British in England and Scotland **(GBR);** Iberian Population in Spain **(IBS));** South Asian (**SAS)- ((**Gujarati Indian from Houston, Texas **(GIH);** Punjabi from Lahore, Pakistan **(PJL);** Bengali from Bangladesh **(BEB);** Sri Lankan Tamil from the UK **(STU);** Indian Telugu from the UK **(ITU))**

### Statistical analyses

The statistical tools available at Metaboanalyst (https://www.metaboanalyst.ca/MetaboAnalyst/ModuleView.xhtml) [40] were used for multivariate principle component analysis (PCA), partial least square- discriminant analysis (PLS-DA) and hierarchical clustering based on the MAFs (defined based on frequencies in global population) of 159 drug-response related SNPs as well as pairwise LD measures (r^2^ and D'). Euclidean distance based Ward’s algorithm was applied in hierarchical clustering to generate population dendrogram. All graphs were generated using the GraphPad Prism^®^ (Version 6) software.

## Results

### Distribution of drug-response related SNPs across populations

**Fig 1:**
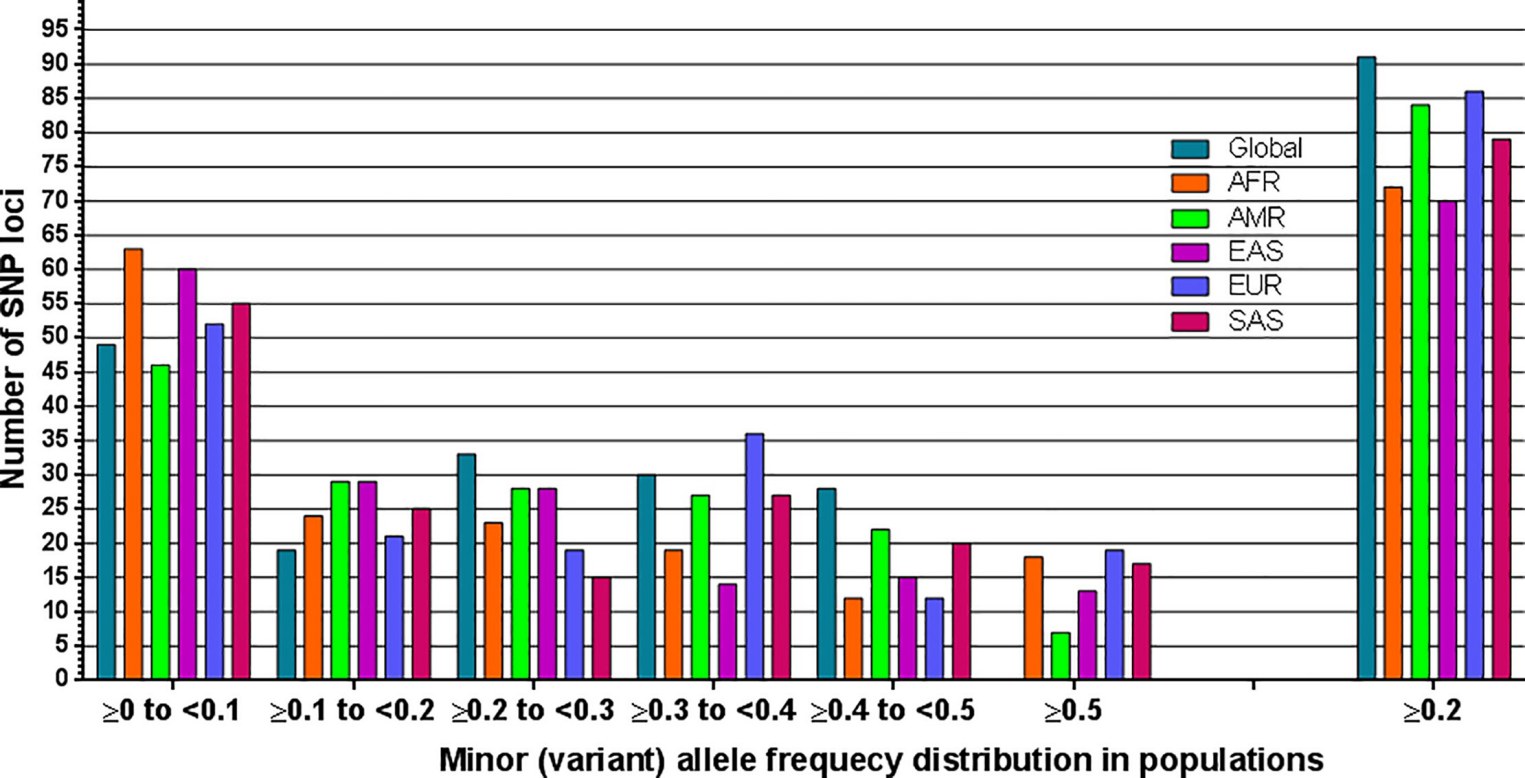
Drug-response related minor (variant) allele frequency distribution in global and five super populations. AFR = African, AMR = Admixed Americans, EAS = East Asian, EUR = European, SAS = South Asian.

We compiled the allele frequencies of 159 drug-response related SNP loci in a total of 32 populations (one global, five super populations and twenty six individual populations) (S1 Table). Defining an allele as minor (frequency <0.5) based on its global distribution may not be always appropriate since globally defined minor alleles may be present as the more prevalent ones in certain populations [41]. 65 of these drug-response related alleles that are considered as minor (variant) in global population are present as the major alleles (frequency ≥0.5) in at least one population (Table 1). In fact, 14 of these drug-response related SNPs have MAFs ≥ 0.8 in at least one of the individual populations. 7 of these SNPs (rs1056836, rs7793837, rs776746, rs2740574, rs6977820, rs1954787 and rs5443) have MAFs ≥ 0.8 only in multiple African sub-populations and 6 (rs2359612, rs8050894, rs9934438, rs9923231, rs7196161 and rs1346268) have MAFs ≥ 0.8 only in several East Asian sub-populations. rs7294 has MAF ≥ 0.8 in only two South Asian populations (STU and ITU). MAFs at majority of the loci show similar distribution patterns among the individual sub-populations within each super-population (Table 1 and S1 Table). The drug-response related allele frequency distribution is different among super-populations indicating demographic effects (Fig 1).

SNPs can be arbitrarily divided into many classes based on their allele frequencies [42, 43]. In this study, we considered frequencies ≥ 0.2 to be comparatively high for the minor (variant) allele at any locus in any population. We observed 111 SNPs which have MAFs ≥ 0.2 in at least one of the super-populations (S1 Table). 31 of these 111 SNPs have MAFs ≥ 0.2 in all super-populations. MAFs at 13 SNP loci are ≥ 0.2 in all twenty-six individual sub-populations. These 13 SNPs are rs2297480, rs6166, rs3812718, rs2952768, rs2228001, rs1902023, rs1042713, rs1042522, rs3212986, rs4680, rs1135840, rs1041983 and rs5443. 18 SNPs have MAFs ≥ 0.2 in only one of the super-populations. These SNPs are rs7582141, rs6432512, rs264588, rs264631, rs2231142, rs7779029, rs2740574, rs6988229, rs885004, rs4917639, rs11045879, rs7297610, rs17708472, rs2884737, rs6065, rs1876828, rs16960228 and rs8099917. 28 SNPs are totally absent (MAF = 0) in all sub-populations belonging to at least any one of the super-populations. 72 SNPs have very low (≤ 0.05) MAFs in at least one of the super-populations. 23 of the drug-response related SNPs have MAF = 0 in majority (>13) of the 26 populations (S1 Table).

Private alleles, which are only present in a particular population among a broader collection of populations, are very useful in population genetics and human evolutionary genetics [44]. We found minor alleles of rs186335453 (T allele) and rs139801276 (C allele) to be private in LWK and all African sub-populations (except ACB), respectively. Minor alleles of rs111033610 (G allele) and rs5030865 (T allele) are private to the East Asian sub-populations (except JPT and CHS, respectively), and the T (variant) allele of rs56019966 is private to 3 European sub-populations (TSI, GBR and IBS).

### LD patterns of the drug-response related SNPs

**Fig 2:**
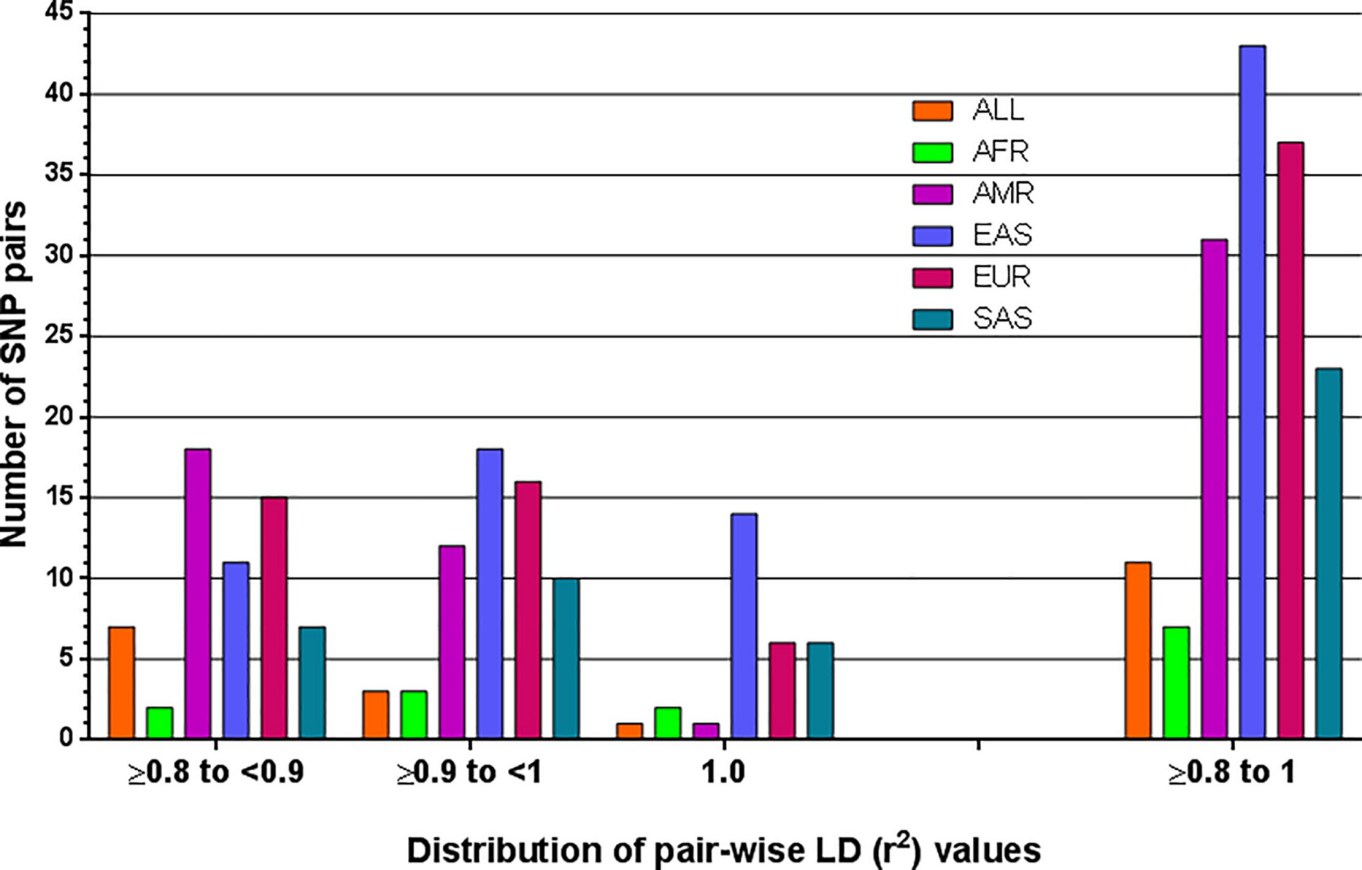
Distribution of pairwise LD (r^2^) values among the drug-response related minor (variant) alleles in global and 5 super populations. AFR = African, AMR = Admixed Americans, EAS = East Asian, EUR = European, SAS = South Asian.

r^2^ and D' are the two most widely used measures of LD. r^2^ is more robust and correlates better among different population samples [45]. We found 48 SNP pairs with r^2^ ≥ 0.8 in at least one of the five super-populations (Table 2). 4 of these pairs have r^2^ values ≥ 0.8 in all super-populations. Interpopulation variability was observed at the levels of LD between drug-response associated SNP loci (Fig 2). 7 SNP pairs with r^2^ ≥ 0.8 are found in African, 31 in Admixed American, 43 in East Asian, 37 in European and 23 are in South Asian super-population (Table 2). East Asian super-population has very strong pairwise LD among 32 SNP pairs (r^2^ ≥ 0.9).

**Table 2:** List of drug-response related SNP pairs with r^2^ ≥ 0.8 in at least one of the five super-populations.

SNP pairs		Chromosome	ALL	AFR	AMR	EAS	EUR	SAS
rs264588	rs7582141	2	***0*.*816***	***0*.*929***	***0*.*979***	***0*.*857***	***0*.*953***	0.239
rs264588	rs6432512	2	0.795	***0*.*880***	***0*.*969***	***0*.*857***	***0*.*953***	0.239
rs264588	rs10497203	2	0.414	0.196	***0*.*802***	***0*.*829***	0.790	0.122
rs264588	rs264651	2	0.564	0.230	***0*.*833***	***0*.*972***	***0*.*859***	***0*.*846***
rs264631	rs7582141	2	0.718	0.686	***0*.*928***	***0*.*870***	***0*.*953***	0.237
rs264631	rs6432512	2	0.699	0.642	***0*.*919***	***0*.*870***	***0*.*953***	0.237
rs264631	rs10497203	2	0.361	0.136	0.760	***0*.*841***	0.790	0.121
rs264631	rs264588	2	***0*.*869***	0.736	***0*.*949***	***0*.*958***	***1*.*000***	***0*.*991***
rs264631	rs264651	2	0.498	0.171	0.790	***0*.*986***	***0*.*859***	***0*.*838***
rs264651	rs10497203	2	0.684	0.714	***0*.*965***	***0*.*854***	***0*.*873***	0.144
rs264651	rs7582141	2	0.387	0.205	***0*.*815***	***0*.*883***	***0*.*808***	0.054
rs264651	rs6432512	2	0.377	0.194	***0*.*807***	***0*.*883***	***0*.*808***	0.054
rs6165	rs6166	2	0.630	0.208	0.783	***0*.*880***	***0*.*992***	***0*.*940***
rs6432512	rs7582141	2	***0*.*976***	***0*.*949***	***0*.*990***	***1*.*000***	***1*.*000***	***1*.*000***
rs6432512	rs10497203	2	0.510	0.204	***0*.*816***	***0*.*972***	***0*.*829***	0.477
rs7582141	rs10497203	2	0.523	0.216	***0*.*825***	***0*.*972***	***0*.*829***	0.477
rs1142345	rs1800460	6	0.318	0.043	0.687	NA	***0*.*965***	0.232
rs1360780	rs4713916	6	0.455	0.080	0.717	0.689	0.699	***0*.*800***
rs713598	rs10246939	7	***0*.*931***	***0*.*970***	***0*.*865***	***0*.*996***	***0*.*855***	***0*.*927***
rs1726866	rs10246939	7	0.799	0.446	***0*.*878***	***0*.*996***	***0*.*992***	***1*.*000***
rs713598	rs1726866	7	0.751	0.443	0.758	***1*.*000***	***0*.*855***	***0*.*927***
rs1208	rs1801280	8	***0*.*823***	0.611	***0*.*904***	***0*.*948***	***0*.*918***	***0*.*914***
rs1799930	rs1041983	8	0.532	0.317	0.504	0.439	***0*.*887***	0.743
rs7853758	rs885004	9	0.565	0.213	***0*.*815***	***0*.*920***	***0*.*909***	0.799
rs4244285	rs12777823	10	***0*.*858***	0.583	***0*.*896***	***0*.*991***	***0*.*939***	***0*.*982***
rs10509681	rs1799853	10	***0*.*850***	***0*.*825***	***0*.*937***	***1*.*000***	***0*.*823***	0.732
rs75838422	rs7900194	10	***1*.*000***	***1*.*000***	***1*.*000***	***1*.*000***	***1*.*000***	***1*.*000***
rs554405994	rs116855232	13	0.217	0.000	***0*.*800***	0.233	0.000	0.000
rs1719247	rs1346268	15	0.537	0.099	***0*.*849***	***0*.*950***	***0*.*946***	0.744
rs9934438	rs2359612	16	***0*.*863***	0.265	***0*.*948***	***1*.*000***	***1*.*000***	***1*.*000***
rs9923231	rs2359612	16	***0*.*862***	0.265	***0*.*943***	***1*.*000***	***1*.*000***	***1*.*000***
rs9923231	rs9934438	16	***0*.*999***	***1*.*000***	***0*.*994***	***1*.*000***	***1*.*000***	***1*.*000***
rs8050894	rs2359612	16	0.646	0.003	***0*.*833***	***1*.*000***	***0*.*951***	***0*.*976***
rs9934438	rs8050894	16	0.774	0.167	***0*.*884***	***1*.*000***	***0*.*951***	***0*.*976***
rs9923231	rs8050894	16	0.774	0.167	***0*.*878***	***1*.*000***	***0*.*951***	***0*.*976***
rs7196161	rs8050894	16	0.720	0.323	***0*.*827***	***1*.*000***	***0*.*899***	***0*.*921***
rs2359612	rs7294	16	0.463	0.181	0.497	***0*.*971***	0.365	0.534
rs8050894	rs7294	16	0.511	0.279	0.525	***0*.*971***	0.379	0.547
rs9934438	rs7294	16	0.400	0.048	0.471	***0*.*971***	0.365	0.534
rs9923231	rs7294	16	0.399	0.048	0.469	***0*.*971***	0.365	0.534
rs7196161	rs7294	16	0.572	0.494	0.506	***0*.*971***	0.368	0.536
rs7196161	rs2359612	16	0.645	0.196	0.773	***1*.*000***	***0*.*852***	***0*.*898***
rs7196161	rs9934438	16	0.551	0.035	0.727	***1*.*000***	***0*.*852***	***0*.*898***
rs7196161	rs9923231	16	0.550	0.035	0.722	***1*.*000***	***0*.*852***	***0*.*898***
rs12979860	rs11881222	19	0.569	0.182	***0*.*879***	***0*.*949***	***0*.*909***	***0*.*845***
rs8099917	rs11881222	19	0.441	0.100	0.636	***0*.*873***	0.463	0.714
rs8099917	rs12979860	19	0.264	0.022	0.566	***0*.*920***	0.428	0.637
rs1065852	rs3892097	22	0.329	0.507	***0*.*855***	0.002	***0*.*903***	0.623

We found 10 haplotypes (2 in chromosome 8, 9 and 19 each, and 1 in chromosome 6, 7, 10 and 16 each) having ≥ 2 variant alleles as well as with frequencies ≥ 0.2 in at least one of the five super-populations (Table 3). All the alleles in the haplotype (T_A_C) on chromosome 7 are minor alleles at the corresponding loci in the global population. This haplotype is present in all five super-populations.

**Table 3:** Haplotypes with frequencies ≥ 0.2 as well as having ≥ 2 variant alleles in at least one of the five super-populations.

Chromosome	SNP ID	Haplotypes*,^#^	Population	Frequency	Length, bp	Associated Drugs
6	rs1142345_ rs1800460_ rs1360780_ rs4713916	T_C***_T_A***	AMR	0.232	17571519	Antidepressants, citalopram, fluoxetine, mirtazapine, paroxetine, selective serotonin reuptake inhibitors, venlafaxine
6	rs1142345_ rs1800460_ rs1360780_ rs4713916	T_C***_T_A***	EUR	0.268		
6	rs1142345_ rs1800460_ rs1360780_ rs4713916	T_C***_T_A***	SAS	0.308		
7	rs10246939_rs1726866_rs713598	***T_A_C***	AFR	0.330	741	Phenylthiocarbamide tasting
7	rs10246939_rs1726866_rs713598	***T_A_C***	AMR	0.284		
7	rs10246939_rs1726866_rs713598	***T_A_C***	EAS	0.323		
7	rs10246939_rs1726866_rs713598	***T_A_C***	EUR	0.538		
7	rs10246939_rs1726866_rs713598	***T_A_C***	SAS	0.638		
8	rs1041983_rs1801280_rs1799930_rs1208	C***_C***_G***_G***	AFR	0.289	521	Ethambutol, isoniazid, pyrazinamide, rifampin
8	rs1041983_rs1801280_rs1799930_rs1208	***T***_T***_A***_A	AFR	0.231		
8	rs1041983_rs1801280_rs1799930_rs1208	C***_C***_G***_G***	AMR	0.356		
8	rs1041983_rs1801280_rs1799930_rs1208	***T***_T***_A***_A	EAS	0.256		
8	rs1041983_rs1801280_rs1799930_rs1208	C***_C***_G***_G***	EUR	0.433		
8	rs1041983_rs1801280_rs1799930_rs1208	***T***_T***_A***_A	EUR	0.281		
8	rs1041983_rs1801280_rs1799930_rs1208	***T***_T***_A***_A	SAS	0.354		
8	rs1041983_rs1801280_rs1799930_rs1208	C***_C***_G***_G***	SAS	0.344		
9	rs7853758_rs885004	***A***_G	AFR	0.240	8624	Anthracyclines and related substances
9	rs7853758_rs885004	***A_A***	AMR	0.200		
10	rs12777823_rs4244285_rs1799853_rs7900194_rs10509681_rs75838422	***A_A***_C_G_G_T	EAS	0.313	2056321	Warfarin, proguanil, mephenytoin, amitriptyline, citalopram, clomipramine, clopidogrel
10	rs12777823_rs4244285_rs1799853_rs7900194_rs10509681_rs75838422	***A_A***_C_G_G_T	SAS	0.357		
16	rs7294_rs2359612_rs8050894_rs9934438_rs9923231_rs7196161	C***_A_G_A_T_G***	AMR	0.383	8660	Warfarin, acenocoumarol, phenprocoumon, vitamin K-dependent clotting factors
16	rs7294_rs2359612_rs8050894_rs9934438_rs9923231_rs7196161	C***_A_G_A_T_G***	EAS	0.885		
16	rs7294_rs2359612_rs8050894_rs9934438_rs9923231_rs7196161	C***_A_G_A_T_G***	EUR	0.372		
19	rs11881222_rs12979860_rs8099917	***G_T***_T	AFR	0.256	8242	Peginterferon alfa-2a, peginterferon alfa-2b, and ribavirin, telaprevir, boceprevir
19	rs11881222_rs12979860_rs8099917	***G_T_G***	AMR	0.275		

*Order of the SNP alleles in the haplotypes are shown in the 2^nd^ (SNP ID) column.

^#^Minor (variant) alleles are shown as bold italic font.

### Geographic distribution of the drug-response related SNPs

**Fig 3:**
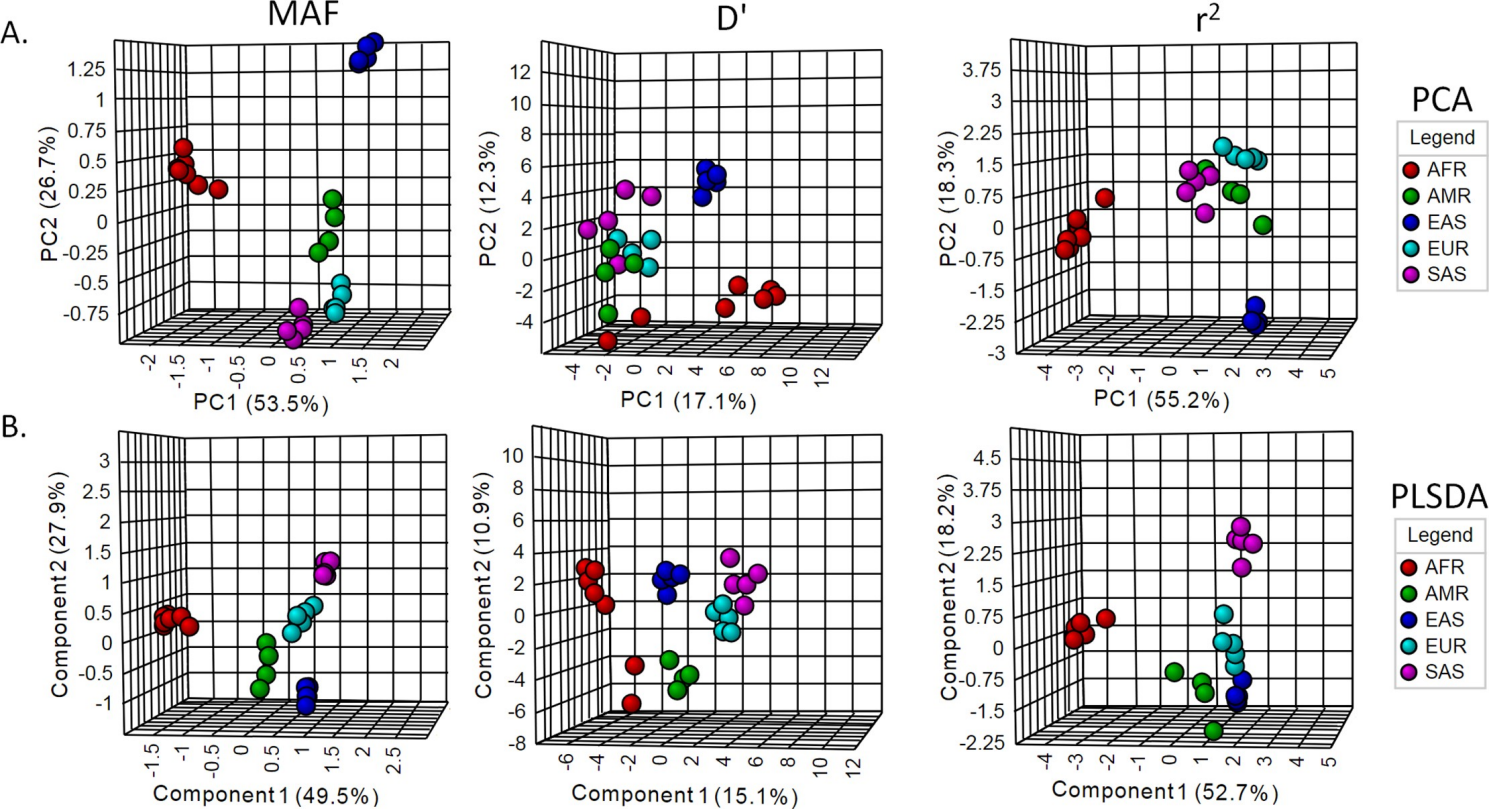
Multivariate analysis using MAF, r^2^ and D' of the drug-response related SNPs in 26 populations. A. Principle component analysis (PCA). B. Partial least square- discriminant analysis (PLS-DA).

**Fig 4:**
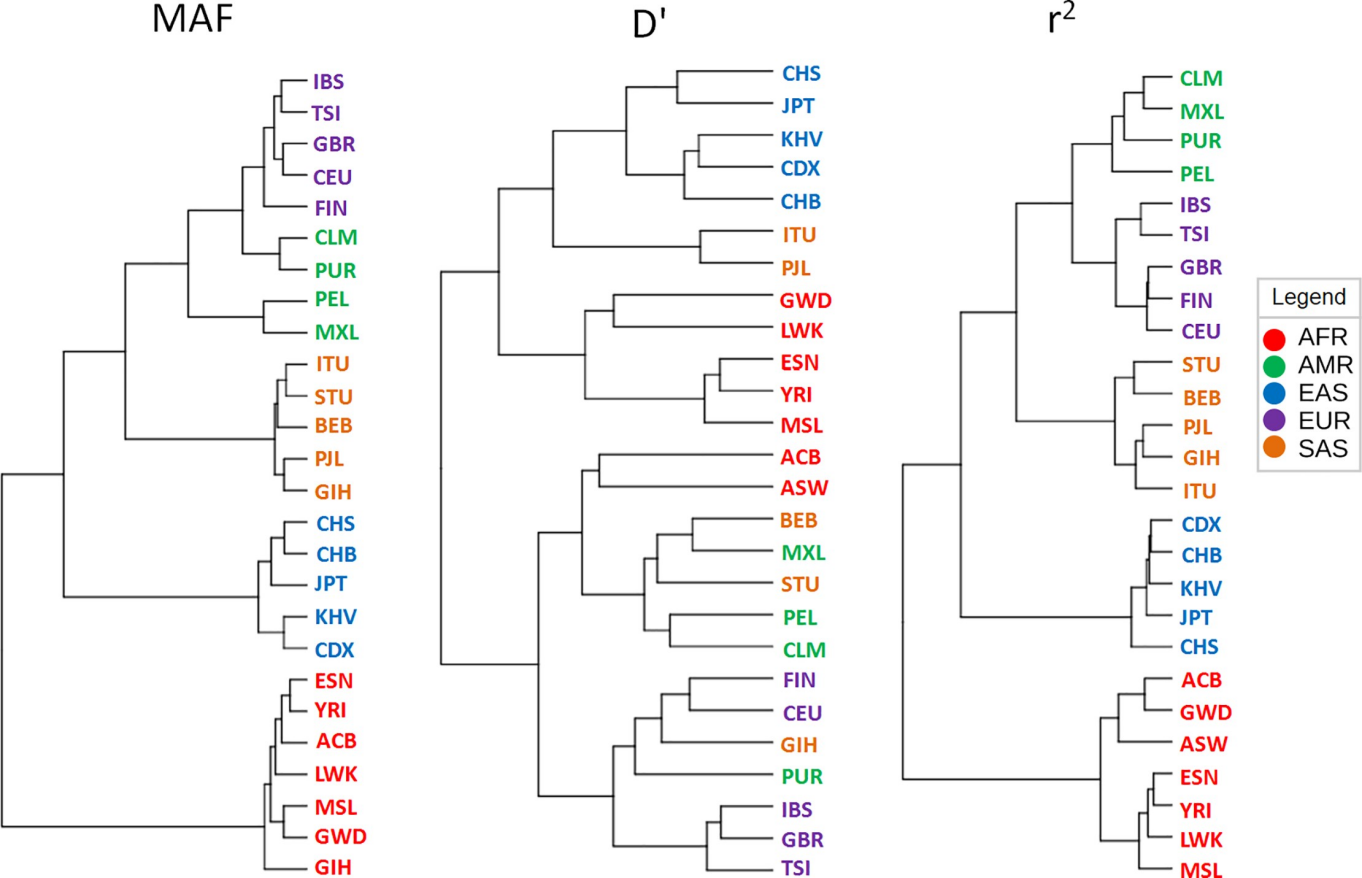
Multivariate analysis using hierarchical clustering. The dendrogram was constructed using the Ward clustering algorithm. The distances are not represented to scale on the tree.

We used MAFs (alleles that are considered as minor in global population) of the 159 SNPs, and both r^2^ and D' estimates of pairwise LD among these SNPs for multivariate clustering through principal component analysis (PCA), partial least square- discriminant analysis (PLS-DA), and hierarchical clustering (Figs 3 and 4). We used the first 2 components in PCA and PLS-DA to visualize the clustering pattern. With MAFs, the 1^st^ and the 2^nd^ components of both PCA and PLS-DA can explain more than 75% of the variations among the sub-populations (Fig 3A and 3B). The 1^st^ and the 2^nd^ components of both PCA and PLS-DA with r^2^ can explain > 70% variations among the populations (Fig 3A and 3B). As shown in the Fig 3A and 3B, component populations of the same super-populations cluster together. Americans of African ancestry in USA (ASW) and the African Caribbeans in Barbados are placed along with the African super-population. Hierarchical clustering of the MAF and r^2^ values using Euclidean distance measure and Ward’s algorithm cluster the component populations of each super-population in a similar way (Fig 4). In both MAF and r^2^ based dendrograms, African populations form a completely different branch from rest of the populations. In the other branch, the East Asian populations form a different clade from the Admixed American, European and South Asian populations. The other LD measure (D') cannot cluster the component populations as distinctively as in PCA and hierarchical clustering (Figs 3A and 4). Although D' places the component populations of super-populations in separate clusters in PLS-DA, their clustering is less obvious than the MAF and r^2^ based plots. Besides, in case of both PCA and PLS-DA using MAF and r^2^, but not D', the 1^st^ component can distinctly separate African population cluster from clusters of other populations (Fig 3A and 3B). It is to be noted that PLS-DA is a supervised multivariate clustering method, which takes into consideration the data classes during the clustering process, while PCA is an unsupervised method.

## Discussion

### Drug-response related SNPs with high MAFs in global population and their clinical importance

Among the 159 drug-response related SNPs, we found 13 SNPs that have MAFs ≥ 0.2 in all super- and sub-populations. These SNPs are responsible for variable responses to bisphosphonates (rs2297480), carbamazepine, phenytoin and antiepileptics (rs3812718), fentanyl, morphine and opioids (rs2952768), cisplatin (rs2228001, rs1042522, rs3212986) oxazepam and lorazepam (rs1902023), salbutamol and salmeterol (rs1042713), antineoplastic agents such as cyclophosphamide, fluorouracil and paclitaxel (rs1042522), platinum and platinum compounds (rs3212986), nicotine (rs4680), debrisoquine (rs1135840), ethambutol, isoniazid, pyrazinamide and rifampin (rs1041983), and sildenafil (rs5443)- drugs that are prescribed for conditions like epilepsy, postmenopausal osteoporosis, pain relief, osteosarcoma, urinary bladder cancer, ovarian cancer, medulloblastoma, brain cancer, breast cancer, neutropenia, stomach cancer, non-small-cell lung carcinoma, colorectal cancer, esophageal cancer, pancreatic cancer, uterine cervical cancer, anxiety, insomnia, asthma, tuberculosis, etc [36, 37, 41]. rs6166 probably renders follicle-stimulating hormone receptor (FSHR) more sensitive to FSH by overcoming feedback inhibition [46]. Flurouracil (a common anti-cancer drug) and nicotine have been frequently reported to exhibit differences in drug response among different populations [8]. High MAF values at rs1042524 (also rs1042522) may play a role in such discrepancies.

One of these (rs2228001) variant-drug combinations has reached level 1B of clinical annotation [37]. Level 1B indicates annotation for a variant-drug combination in Clinical Pharmacogenomics Implementation Consortium (CPIC) or medical society-endorsed Pharmacogenomics (PGx) guideline, or implemented at a Pharmacogenomics Research Network (PGRN) site or in another major health system, for which the preponderance of evidences show an association. Patients with GG or GT genotype at rs2228001 may have an increased risk of cisplatin toxicity in comparison with those with TT genotype [37]. Another interesting variant-drug combination is rs4680-nicotine, which has level 2A clinical annotation evidence. The variants in level 2A are located in known pharmacogenes, and therefore, functional significance is more likely. rs4680 is located in the COMT gene. Individuals with the AA or AG genotype at rs4680, who are treated with nicotine replacement therapy (NRT), may have an increased likelihood of smoking cessation and decreased risk of relapse as compared to individuals with the GG genotype. Although A is the global minor allele at rs4680 (Table 1), its frequency is ≥ 0.5 in FIN, GBR and PJL sub-populations. The long term benefit of NRT is actually the requirement of modest and repeated episodes of such treatment [47]. Differences in the efficacy of NRT between men and women have been reported as well. Gains from long-term NRT decrease more rapidly for women than men [48]. Genotyping at the rs4680 locus may be considered while assessing the factors influencing NRT efficacy for the treatment of tobacco use disorder.

### Drug-response related SNPs with high MAFs in individual populations and their clinical importance

18 SNPs have MAFs ≥ 0.2 in only one super-population (Table 1). 11 of these have MAFs ≥ 0.2 in African super-population. These are responsible for variable response to radiotherapy for prostate neoplasm (rs7582141, rs6432512, rs264588 and rs264631), irinotecan (rs7779029), tacrolimus (rs2740574), salbutamol (rs6988229), warfarin (rs4917639), hydrochlorothiazide (rs7297610 and rs16960228) and aspirin (rs6065). Four of these SNPs (rs7582141, rs6432512, rs264588 and rs264631) may be associated with variable risk of toxicity in response to radiotherapy for prostate neoplasm. There is level 2B clinical annotation evidence for these four variant-drug combinations [37]. Although data on prostate cancer treatment in Africa is under-reported [49], it is known that African men disproportionately suffer from prostate cancer compared to men from other parts of the world [50] and African American men have the highest rate of prostate cancer morbidity and mortality compared to men from any other race or ethnicity in the USA [51]. Socioeconomic and genetic factors are among the suggested explanations for such high burden of prostate cancer in African men [52]. There is no evidence that prostate cancer in African Americans is more virulent than in Caucasians [53]. But there are population-level genetic differences in androgen receptor signaling and DNA repair between African American and Caucasian men’s prostate cancer and African American men may harbor more radiosensitive tumors, which may result in better clinical outcomes from radiotherapy in African American patients with prostate cancer [54]. Since further studies are needed to conclusively find out all the factors affecting the efficacy of radiotherapy in African prostate cancer patients, the risk of increased toxicity of radiotherapy in prostate cancer patients with certain genotypes at rs7582141, rs6432512, rs264588 and rs264631 and their high (≥0.2) MAFs in African super-population should be considered. A discrete screening guideline may be helpful in treating African American men with prostate cancer [55] along with a distinct clinical guideline for radiotherapy.

The minor allele (C) at the rs4917639 locus- (located in CYP2C9 gene) is present with ≥0.2 frequency only in the African super-population. Individuals with the CA or CC genotype may require decreased dose of warfarin compared to those with the AA genotype and there is level 2A clinical annotation for this variant-drug combination [37]. An ethnicity-dependent CPIC guideline for warfarin dosing recommends a dose reduction of 10–25% in African Americans with AG or AA genotype at rs12777823, but not in patients with non-African ancestry [37]. Frequency of the A allele at rs12777823 is 0.251 in African super-population (Table 1). We did not find high pairwise LD between these 2 SNPs in African super-population. So, incorporation of rs4917639 into clinical guidelines may benefit individuals of African ancestry.

rs885004 and rs8099917 have MAFs ≥ 0.2 only in Admixed American super-population. These may cause variable response to anthracyclines and related substances (rs885004) and peginterferon alfa-2a, peginterferon alfa-2b, and ribavirin, telaprevir, boceprevir (rs8099917). There is level 1B clinical evidence for rs8099917 associated variable efficacy of peginterferon alfa-2a, peginterferon alfa-2b, ribavirin, telaprevir and boceprevir in chronic Hepatitis C treatment [37]. So, patients belonging to Admixed American super-population may benefit from dosing guidelines for these drugs based on the rs8099917 genotypes. In fact, it has been suggested that at least in HCV infected Caucasian patients simultaneous genotyping of rs12979860 and rs8099917 should be recommended prior to the initiation of pegylated interferons and ribavirin treatment [56]. The global minor allele (T) at rs12979860 has a frequency of 0.399 in Admixed American super-population (S1 Table). Determination of the rs8099917 genotype may benefit a significant proportion of heterozygous carriers of the rs12979860 T non-responder allele with respect to sustained virologic response prediction [57].

Two SNPs (rs2231142 and rs11045879) have MAFs ≥ 0.2 only in the East Asian super-population (Table 1). These may cause variable response to rosuvastatin and allopurinol (rs2231142) and methotrexate (rs11045879). There is level 2A clinical annotation for all these variant-drug combinations [37]. Ethnic differences in response to rosuvastatin (especially, the increased systemic exposure to this drug in people with Chinese ethnicity) have been mentioned earlier. FDA recommends Asian patients to initiate rosuvastatin at half of the normal dose for non-Asians [58].

rs17708472, rs2884737 and rs1876828 have MAFs ≥ 0.2 only in the European super-population. These SNPs may be responsible for variable response to warfarin (rs17708472 and rs2884737, which are located in VKORC1) and budesonide, corticosteroids, fluticasone propionate, fluticasone/salmeterol and triamcinolone (rs1876828). Genotype at rs17708472 and rs2884737 may influence warfarin dose requirement [37]. There is level 2A clinical evidence for the variant-drug combinations for these two SNPs [37]. Genotype at rs1876828 may affect the efficacy and, therefore, response to inhaled corticosteroids may influence resulting endogenous cortisol level [37]. rs1876828 is located in CHR1 gene, which is targeted with drugs to treat asthma. There is level 2B clinical annotation for these variant-drug combinations [37]. There is currently no clinical guideline for inhaled cortecosteroids that are used to treat asthma.

5 SNPs (rs2359612, rs8050894, rs9934438, rs9923231 and rs7196161) have MAF ≥ 0.8 in all East Asian sub-populations. These share absolute LD (r^2^ = 1) among them (S1 Fig). These SNPs cause variability in response to warfarin (rs2359612, rs8050894, rs9934438, rs9923231 and rs7196161), acenocoumarol and phenprocoumon (rs9934438 and rs9923231), and vitamin K-dependent clotting factors (rs9934438) [35,36]. As discussed earlier, there is level 1A clinical annotation for rs9923231-warfarin combination and level 1B clinical annotation for rs9934438-warfarin, rs9923231-acenocoumarol, and rs9923231-phenprocoumon combinations [37]. Individuals with CT genotype at rs9923231 may require a decreased dose of warfarin, acenocoumarol and phenprocoumon as compared to those with the CC genotype or an increased dose as compared to those with TT genotype [37]. Individuals with AA genotype at rs9934438 may require a lower dose of warfarin as compared to patients with the AG or GG genotype [37]. Chinese patients require lower dose of warfarin than Caucasian patients and VKORC1 genotype has already been suggested to be an important determinant of warfarin response in Chinese patients [59]. The same study reported the high frequencies (≥0.8) of the global minor alleles at rs9923231 and rs9934438 loci in Chinese population. So, reduced dosage of warfarin, acenocoumarol and phenprocoumon for individuals from East Asian populations may be recommended. High frequency of T allele at rs9923231 in East European populations may be the result of positive selection [60]. The absolute pairwise LD among rs2359612, rs8050894, rs9934438, rs9923231 and rs7196161 in East Asian populations is not an unusual finding. In fact, a 505 kb region of strong LD, which contains VKORC1 and 24 neighboring genes, is located on chromosome16 only in East Asian populations and this genomic region may have been submitted to a near complete selective sweep in all East Asian populations and only in this geographic area [61].

rs1954787 has MAF ≥ 0.8 in all African sub-populations and is responsible for variable response to antidepressants. Currently, there is level 2B clinical annotation for this variant-drug combination. Individuals with CT or TT genotype and depressive disorder or depression may be less likely to respond to antidepressant treatment as compared to those with CC genotype [37]. Major depressive disorder (MDD) usually remains untreated and is more severe and disabling in the African Americans and Caribbean Blacks compared with Anglo Americans [62]. Consequently, the burden of mental disorders, especially depressive disorders, may be higher in African Americans [62]. If the association between genotype at rs1954787 and variable response to antidepressants becomes strongly definitive, this marker may be employed in conjunction with other known predictors to anticipate the outcome of treatments with antidepressants [63] considering the fact that more than 80% patients with African ancestry may be less likely to respond to antidepressants.

In addition to these, level 1A clinical annotation is available for the following variant-drug combinations: rs887829-atazanavir; rs1142345-azathioprine, mercaptopurine, purine analogues, thioguanine; rs1800460-azathioprine, mercaptopurine, purine analogues, thioguanine; rs12248560-clopidogrel; rs28399504-clopidogrel; rs4986893- clopidogrel; rs1057910-warfarin; rs4149056-simvastatin; rs116855232-azathioprine, mercaptopurine; rs9923231- warfarin; and rs12979860-peginterferon alfa-2a, peginterferon alfa-2b, ribavirin [37]. Level 1B clinical annotation is available for the following variant-drug combinations and rs3745274-efavirenz [37].

### SNPs in the Cytochrome P450 genes

Cytochrome P450 family genes (*CYP*) have been extensively studied in the context of pharmacogenomics because of their important roles in drug metabolism [64, 65]. Ethnic differences in these genes have been reported [14]. MAFs of *CYP* genes in all the super-populations are listed in S2 Table. Among these rs1135840, rs16947, rs1065852, rs12248560, rs4244285, rs4917639, rs3745274, rs776746, rs2740574, rs25487, rs2108622 and rs1056836 have MAF ≥ 0.2 in multiple super-populations. Level 1A clinical annotation is available for rs2108622-warfarin combination. Individuals with TT genotype at rs2108622 may require a higher dose of warfarin as compared to those with CC or CT genotype [37]. African populations stand out different from the other populations in terms of other SNPs in CYP genes as well. African populations are known to have different frequencies of certain ADRs than rest of the world [66]. The major alleles (frequency ≥ 0.5) at the rs16947, rs776746, rs2740574 and rs1056836 loci in most African subpopulations are actually global minor alleles. There are level 1A clinical annotations available for rs16947 (an SNP defining CYP2D6*2 allele)- paroxetine, nortriptyline, codeine, doxepin, trimipramine, clomipramine, atomoxetine and amitriptyline and rs776746- tacrolimus combinations [37]. T is the global minor allele at rs776746 and recipients of kidney, heart, lung or hematopoietic stem cell transplant, who have CT or TT genotype at rs776746 may require a higher dose of tacrolimus compared to those with CC genotype [37]. Differences in the allele frequencies at rs776746 between the European descendant and the African American individuals is partly responsible for the lower trough blood concentration of tacrolimus in African American kidney allograft recipients compared to the European descendants [67]. An African American-specific genotype-guided tacrolimus dosing model has recently been developed since African Americans have 20–50% lower bioavailability, higher clearance and lower blood concentration of tacrolimus and, as a result, require ~1.5–2 times higher doses than the Caucasians [68]. Other African populations may also benefit from this guideline. On the other hand, aroxetine, trimipramine, atomoxetine, clomipramine and amitriptyline are used to treat various mental disorders, especially depressive disorder [37]. It again shows the difficulty in selecting an efficacious drug to treat mental disorders in patients with African ancestry. There is level 1B clinical annotation for rs16947 (an SNP defining CYP2D6*2 allele)-tramadol combination. So, tramadol and codeine, both of which are used to treat pain, may have less than optimum response in the majority of individuals with African ancestry. It may have serious clinical implications as there are racial/ethnic disparities in pain epidemiology, access to quality pain care, pain assessments and treatments and pain-related outcomes [69]. rs16497 can reduce CYP2D6 expression by about 2 folds and thus may reduce overall CYP2D6 metabolic activity [70]. Incorporation of rs16947 along with another SNP into *CYP2D6* biomarker panel may improve the accuracy of CYP2D6 metabolizer status prediction [71]. Poor metabolizers with less CYP2D6 activity may have very little analgesic efficacy for codeine [72]. Codeine is often prescribed to individuals with sickle cell disease (SCD) and precision medicine approach is necessary to maintain it as a safe option for pain control [73]. SCD is very common throughout much of sub-Saharan Africa [74]. African Americans with SCD are less genetically admixed than other African Americans and have an ancestry similar to Yorubans, Mandinkas and Bantu [75]. So, SCD may be more prevalent among individuals that are more closely related to sub-Saharan Africans. Moreover, the only two African sub-populations with MAF < 0.5 at rs16947 are ASW and ACB (Table 1). The sub-Saharan African populations have MAF ≥ 0.5 at rs16947. So, codeine may be less likely to be effective in individuals closely related to sub-Saharan Africa. Hence, alternative drugs may be considered for managing pain in SCD patients with African ancestry. Although we did not find extensive *CYP* allele frequency variations among the African populations as reported in a previous study [76], our results also emphasize the need for the population targeted optimization and development of drugs.

### Drug-response related SNP haplotypes with high frequencies

Apart from the 10 haplotypes with at least two global minor alleles and frequencies ≥ 0.2 in at least one super-population (Table 3), there is an important haplotype (T_T_C_T_T_G_A_G) in the East Asian populations on chromosome 2. Although the frequency of this haplotype (0.1052) is < 0.2, all alleles except the first one are the minor (variant) alleles at the corresponding SNP loci (rs6166_ rs6165_ rs10497203_ rs7582141_ rs6432512_ rs264651_ rs264588_ rs264631) in the global population. All of these SNPs, except rs6166 and rs6165, are responsible for variable responses to radiotherapy for prostate cancer. However, currently none of these variant-drug combinations qualifies for level 1A or level 1B clinical annotation [37]. SNPs in other haplotypes (Table 3) with high prevalence cause variability in response to antidepressants, citalopram, fluoxetine, mirtazapine, paroxetine, selective serotonin reuptake inhibitors, venlafaxine (chromosome 6: T_C***_T_A***), phenylthiocarbamide tasting (chromosome 7: ***T_A_C***), ethambutol, isoniazid, pyrazinamide, rifampin (chromosome 8: C***_C***_G_G and ***T***_T***_A***_A), anthracyclines and related substances (chromosome 9: ***A_A***), warfarin, proguanil, mephenytoin, amitriptyline, citalopram, clomipramine, clopidogrel (chromosome 10: ***A_A***_C_G_G_T), warfarin, acenocoumarol, phenprocoumon, vitamin K-dependent clotting factors (chromosome 16: C***_A_G_A_T_G***) and peginterferon alfa-2a, peginterferon alfa-2b, and ribavirin, telaprevir, boceprevir (chromosome 19: ***G_T***_T and ***G_T_G***) [36, 37]. It is worth noting that multiple SNPs in the haplotype on chromosome 10 are located in *CYP2C* gene region and all except one SNP in the haplotype on chromosome 16 are located in *VKORC1* gene region. These are two very important pharmacogenes. Both of these haplotypes (***A_A***_C_G_G_T and C***_A_G_A_T_G***, respectively) are present in global population with frequency ≥ 0.2. Clinical annotations for rs12777823-warfarin and rs9923231-warfarin combinations have already been discussed. Among the other SNPs in the haplotype on chromosome 10, level 1A clinical annotation is available for rs4244285-clopidogrel, rs4244285-amitriptyline and rs1799853-warfarin combinations [37]. On chromosome 16, level 1B clinical annotation is available for rs7294-warfarin, rs9934438-warfarin and rs9923231-acenocoumarol, phenprocoumon combinations [37].

### LD patterns of the drug-response related SNPs across populations

Presence of long stretches of genomic regions with high LD in a particular population means that a number of neighboring SNPs are in strong or absolute pairwise LD with the functional or causal variant within that population. So, SNPs that are in strong or absolute pairwise LD with the causal variant will give similarly strong association signal. In that case, trans-population analysis, which utilizes differences in LD patterns across different populations, can be used to narrow the list of possible causal variants [77]. Hence, it is important to know the inter-population variability in LD pattern.

Extent of LD is lower in African in comparison to non-African populations [31, 78–84]. We found only 7 SNP pairs with strong pairwise LD (r^2^ ≥ 0.8) in African super-population, compared to 11 pairs in global population (Table 2). 4 SNP pairs were found to have strong LD (r^2^> 0.9) in all super-populations (Table 2). Among the individual super-populations, East Asian had the highest number of SNP loci (43) that maintain strong LD (r^2^≥0.8) with one another. Majority of these SNP pairs (32) maintain r^2^ ≥ 0.9, which is highly distinctive of the East Asian population (Fig 2). It is known that populations with higher extent of LD or background LD are more suitable for initial mapping in GWAS, whereas populations with lower level of LD or background LD are more suitable for subsequent fine mapping of causal variants [5, 85]. So, East Asian population might be investigated for initial mapping in future GWAS for pharmacognomic investigation.

### Human evolution and geographic distribution of the drug-response related SNPs

Multiple studies have used allele frequencies for inferring human population structure [86–88]. We used the MAFs and pairwise LD measures (r^2^ and D') of 159 drug response-related SNPs for multivariate analysis using PCA, PLS-DA and hierarchical clustering (Figs 3 and 4). African populations appear completely distinct from the other populations. Similar results were obtained in previous studies using SNP loci, *Alu* insertion sites and D1S80 allele frequencies [86–89]. In these studies, the East Asian populations appear to be more distant from South Asian, European and Admixed American populations.

Fossil and genetic evidences suggest that anatomically modern humans evolved in Africa about 150,000 to 190,000 years ago and then migrated into Europe, Asia, and finally to the Americas in an approximately West-to-East pattern [82, 90, 91]. Geographic isolation, interbreeding, and adaptation in new environments differentiated human populations from each other [82]. Consistent with the out-of-Africa model of human origin, the Africans possess the oldest genetic pool and the highest level of genetic diversity [92]. Therefore, extent of LD is lower in African in comparison to non-African populations [31, 78–84]. There is more Neanderthal admixture into East Asian populations than into European populations and some extent of admixture occurred after the separation of East Asians and Europeans [93–97]. European and South Asian populations have been reported to be closely related in multiple studies [88, 89, 98]. South Asian populations also share Denisovan ancestry with the East Asians [96]. PCA, PLS-DA and dendrogram plotted with MAF and r^2^, but not D', of drug-response related SNPs could reproduce the human evolutionary history and geographic distribution.

Linkage disequilibrium (LD) that exists among DNA variants in the current human genome is the result of historical evolutionary forces, particularly finite population size, mutation, recombination rate, and natural selection [99]. LD between genetic variants is commonly measured as r^2^ (a squared correlation) or D′ (which is equal to D normalized by its maximum given the allele frequencies) [99, 100]. Though r^2^ or D' both depend on the allele frequencies, r^2^ is a more stringent measure and depends more on allele frequencies [101–103].

In PCA, PLS-DA and dendrogram with MAF and r^2^ values, Americans of African Ancestry (ASW) and African Caribbeans in Barbados (ACB) clustered with the African sub-populations. Based on the historical records, the African Americans and the African Caribbeans in Barbados are descended from slaves who were imported mostly from West Africa during the eighteenth century [27]. African Americans have genetic admixture with approximately 80% of their genome derived from their African ancestors and 20% from the Europeans [12, 82]. Among the African populations in this study, Mende (MSL) and Gambian (GWD) share mostly the Western African ancestry, Esan (ESN) and Yoruba (YRI) peoples from Nigeria share the West-Central African ancestry and the Luhya (LWK) people from Kenya belong to the Bantu-speaking Eastern African ancestry [104]. Since, the slaves in America and Carribeans were brought mostly from West Africa [27], they are supposed to carry more genetic similarity to the African populations than the Southern American ones [104, 105]. As shown in Figs 3 and 4, ASW and ACB populations form a distinct cluster with the other African populations, rather than with Admixed American populations. Admixed American populations appeared more closely genetically related with European populations in dendrogram with both MAF and r^2^.

Latin American populations- Colombia, Mexico, Peru, and Puerto Rico- have distinct patterns of continental genetic admixture [91]. Puerto Rico and Colombia are characterized by substantial ancestry contributions from African, European and Native American groups, whereas Mexico and Peru have primarily Native American and European ancestry [91]. Puerto Rico and Colombia inherited more genetic content from the European ancestry than Peru and Mexico [91, 106–108]. In the MAF based dendrogram, CEL and PUR form a closer branch with the European populations. Such finer distinctions were achieved with MAF and not r^2^. A dendrogram recapitulates the relationships among population groups. Individuals who cluster near each other in the tree could either share a recent common ancestry and/or experienced gene flow [84]. Finnish in Finland (FIN) are estimated to have obtained ~7% of their ancestry from East Asians and admixed American populations, whose Native American ancestors are related to East Asians [96]. MAF based dendrogram could figure out such finer genetic distinctions, which could not be detected with r^2^. So, human migration patterns and demographical history can be more accurately reconstructed with allele frequencies than pairwise LD measures. More dependency of r^2^ on allele frequencies in comparison to D' may explain why r^2^ is better than D' at reconstructing such patterns and history.

Although most of the large inter-continental differences in allele frequency may not result from positive selection [109], there may be numerous cases of recent positive selection of pharmacogenes [110]. We observed many drug-response related SNPs with higher MAFs in certain super- or subpopulations compared to other populations (S1 Table). There is evidence of natural selection for some of these SNPs, while the others require further investigations. For example, among the SNPs with MAFs ≥ 0.8 in at least one super- or subpopulation the possibility of natural selection has been suggested for rs776746 [111], rs2740574 [111], rs2359612 [61], rs8050894 [61], rs9934438 [61], rs9923231 [61], rs1346268 [112], rs7294 [61]. Hence, the pattern of allele frequency distribution observed for these 159 drug response-related SNPs may not be observed for any random 159 SNPs. Many of the SNPs chosen for this study may be under natural selection.

### Can these findings be generalized?

Considering the high similarities of MAFs in sub-populations belonging to the same super-population, it may seem tempting to study only the super-populations to predict drug responses in all of its sub-populations. But such generalizations may not be appropriate. Only 26 sub-populations were included in this study and most of these samples cannot be considered representatives of their source populations. There can be marked differences in the allele frequencies of important pharmacogenes among the sub-populations belonging to the same super-population. Such differences were observed in the allele frequencies of *CYP* genes in African populations [76]. There may be large allele frequency differences even among groups of the same population. This phenomenon is observed in India where endogamy has maintained signatures of strong founder effects for thousands of years [113]. So, different Indian groups may show quite different drug responses. For example, there is a very high frequency of homozygous silent butyrylcholinesterase (BChE) in Vysya community of India [114]. Individuals with this particular genetic variant (BChE L307P) may have negligible activity due to its structural instability as compared to other BChE variants [115]. Administration of muscle relaxant succinylcholine to individuals carrying BChE variants with no or reduced activity may cause prolonged apnea [116]. Deficiency of BChE activity may also cause apnea after administration of neuromascular blocking drug mivacurium [117]. Furthermore, even two neighboring populations living in the same country may have differences in drug response if they have different ancestry or different level of admixture. For example, there are differences in allele frequencies of drug response-related SNPs between two neighboring Colombian populations- Antioquia and Chocó- owing to their distinct ancestry profiles [118].

In our study, we looked at only the SNPs that may cause variable drug responses. But other factors e.g., diet, chemical exposures from the environment, disease state, etc may be sources of variability in drug response as well [37, 119]. Epigenetic modulations of ADME genes and drug targets may be important determinant of responses to drugs [120]. Especially, epigenetics can play an important role in the acquired resistance to chemotherapy in cancer patients and epigenetic biomarkers may predict the outcomes of chemotherapy [121, 122]. In addition to drug-gene interactions, drug-gene-drug interactions may also cause differences in drug response and should be considered during prescribing drugs [123, 124].

## Conclusion

There is a global concern to increase pharmacogenetic testing to ensure drug safety and enhance drug efficacy [125, 126]. However, most GWAS to identify drug-response related variants have been performed in the western populations and others have lagged behind [7, 127, 128]. It is important to understand the interpopulation or interethnic variability in drug response so that population/ethnicity-specific guidelines can be produced. Besides, knowing the SNP distribution and LD patterns of different populations will be helpful in causal variant discovery. In this study, we looked at the interpopulation similarities as well as differences in drug-response related minor (variant) allele frequencies, LD patterns and haplotype distributions. This study may be useful in comparative and evolutionary pharmacogenomics studies among populations in future.

## Supporting information

S1 Fig LD (r^2^) among rs2359612, rs8050894, rs9934438, rs9923231 and rs7196161 in East Asian populations. (TIF)

S1 Table List of 159 drug-response related minor (variant) allele frequency distribution worldwide populations. (PDF)

S2 Table MAFs of *CYP* genes in the super-populations. (DOC)
