# Supplementary figures and images for: Heterogeneity in the distribution of 159 drug-response related SNPs in world populations and their genetic relatedness
Source: PLoS One. 2020 Jan 23;15(1):e0228000. doi: 10.1371/journal.pone.0228000 (PMC6977754; doi:10.1371/journal.pone.0228000)

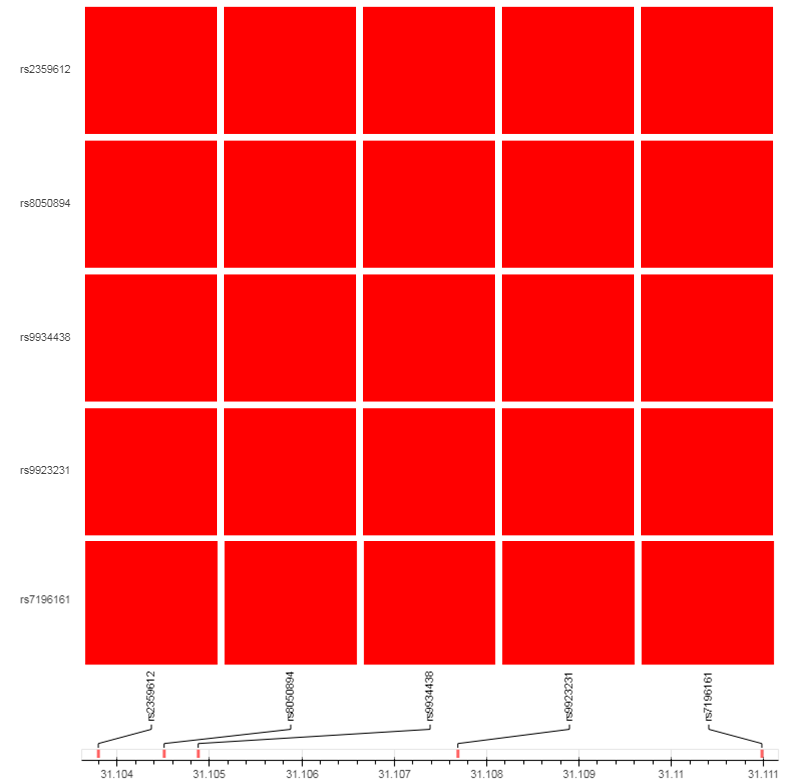

Supplement: S1 Fig — (TIF) [file pone.0228000.s001.tif]
