# Supplementary material for: Heterogeneity in the distribution of 159 drug-response related SNPs in world populations and their genetic relatedness
Source: PLoS One. 2020 Jan 23;15(1):e0228000. doi: 10.1371/journal.pone.0228000 (PMC6977754; doi:10.1371/journal.pone.0228000)
Supplement: S1 Table — (PDF) [file pone.0228000.s002.pdf]

S1 table: Minor (variant) allele frequency* distribution in populations.

			ALL	AFR								AMR					EAS						EUR						SAS					
#	SNP ID	Global minor allele	All pops	AFR	YRI	LWK	GWD	MSL	ESN	ASW	ACB	AMR	MXL	PUR	CLM	PEL	EAS	CHB	JPT	CHS	CDX	KHV	EUR	CEU	TSI	FIN	GBR	IBS	SAS	GIH	PJL	BEB	STU	ITU
1	rs1801131	G	0.249	0.151	0.12	0.187	0.115	0.135	0.136	0.205	0.188	0.151	0.164	0.216	0.133	0.082	0.219	0.223	0.178	0.157	0.29	0.258	0.313	0.082	0.313	0.318	0.341	0.271	0.417	0.417	0.417	0.413	0.372	0.466
2	rs1801133	A	0.245	0.09	0.106	0.071	0.062	0.076	0.081	0.139	0.115	0.474	0.469	0.447	0.543	0.435	0.296	0.466	0.38	0.286	0.134	0.192	0.365	0.435	0.467	0.273	0.324	0.444	0.119	0.15	0.135	0.122	0.083	0.103
3	rs3753380	T	0.218	0.011	0	0.005	0	0	0	0.074	0.026	0.255	0.242	0.231	0.271	0.277	0.322	0.277	0.37	0.295	0.323	0.348	0.324	0.277	0.318	0.414	0.214	0.336	0.253	0.272	0.313	0.25	0.201	0.23
4	rs2297595	C	0.057	0.031	0.009	0.101	0.026	0.012	0.005	0.049	0.021	0.063	0.07	0.086	0.064	0.029	0.018	0	0.01	0.005	0.038	0.04	0.119	0.029	0.107	0.197	0.082	0.117	0.061	0.034	0.109	0.052	0.054	0.059
5	rs2072661	A	0.265	0.282	0.324	0.273	0.19	0.324	0.343	0.303	0.24	0.238	0.188	0.245	0.175	0.335	0.318	0.316	0.264	0.305	0.382	0.328	0.267	0.335	0.234	0.303	0.319	0.243	0.203	0.223	0.167	0.192	0.275	0.152
6	rs2297480	G	0.369	0.282	0.278	0.288	0.288	0.282	0.273	0.328	0.255	0.305	0.383	0.279	0.277	0.312	0.69	0.762	0.736	0.748	0.656	0.535	0.263	0.312	0.21	0.298	0.319	0.224	0.312	0.252	0.344	0.267	0.343	0.348
7	rs1801274	G	0.442	0.526	0.523	0.485	0.482	0.612	0.596	0.492	0.495	0.451	0.508	0.452	0.394	0.471	0.278	0.335	0.192	0.3	0.285	0.278	0.511	0.471	0.406	0.54	0.61	0.528	0.419	0.408	0.391	0.36	0.475	0.451
8	rs1051740	C	0.313	0.141	0.097	0.197	0.097	0.159	0.116	0.172	0.172	0.32	0.438	0.236	0.308	0.347	0.482	0.432	0.481	0.471	0.495	0.535	0.304	0.347	0.29	0.298	0.357	0.29	0.377	0.349	0.391	0.39	0.358	0.402
9	rs2234922	G	0.215	0.353	0.417	0.333	0.327	0.353	0.348	0.295	0.375	0.143	0.117	0.188	0.17	0.076	0.118	0.097	0.125	0.124	0.097	0.146	0.164	0.076	0.164	0.167	0.159	0.136	0.234	0.199	0.276	0.215	0.27	0.211
10	rs1056836	C	0.385	0.817	0.875	0.788	0.823	0.865	0.838	0.697	0.786	0.277	0.305	0.375	0.266	0.147	0.091	0.087	0.106	0.081	0.07	0.111	0.398	0.429	0.402	0.273	0.407	0.472	0.169	0.189	0.167	0.169	0.147	0.172
11	rs6166	C	0.407	0.405	0.481	0.374	0.35	0.418	0.429	0.426	0.365	0.422	0.336	0.476	0.436	0.406	0.322	0.296	0.341	0.309	0.344	0.323	0.449	0.414	0.463	0.5	0.44	0.43	0.445	0.505	0.464	0.355	0.436	0.451
12	rs6165	T	0.492	0.237	0.227	0.222	0.203	0.176	0.247	0.377	0.26	0.579	0.672	0.476	0.558	0.659	0.66	0.67	0.644	0.667	0.667	0.651	0.549	0.586	0.533	0.5	0.56	0.565	0.544	0.49	0.516	0.628	0.554	0.544
13	rs11676382	G	0.026	0.002	0	0	0	0	0	0.016	0.005	0.026	0.016	0.034	0.037	0.012	0	0	0	0	0	0	0.094	0.131	0.042	0.056	0.159	0.093	0.012	0.01	0.021	0.023	0.01	0
14	rs10497203	C	0.094	0.115	0.125	0.151	0.128	0.082	0.121	0.123	0.068	0.141	0.141	0.082	0.165	0.188	0.166	0.117	0.159	0.191	0.161	0.202	0.039	0.025	0.028	0.081	0.028	0.033	0.017	0.01	0.021	0.041	0.01	0.01
15	rs7582141	T	0.155	0.315	0.315	0.298	0.438	0.4	0.247	0.271	0.213	0.163	0.156	0.115	0.186	0.2	0.17	0.117	0.173	0.191	0.167	0.202	0.042	0.025	0.042	0.081	0.028	0.033	0.036	0.015	0.021	0.052	0.059	0.034
16	rs6432512	T	0.159	0.327	0.324	0.308	0.456	0.4	0.273	0.279	0.219	0.164	0.156	0.115	0.192	0.2	0.17	0.117	0.173	0.191	0.167	0.202	0.042	0.025	0.042	0.081	0.028	0.033	0.036	0.015	0.021	0.052	0.059	0.034
17	rs264651	G	0.107	0.1	0.106	0.131	0.115	0.065	0.101	0.123	0.057	0.137	0.125	0.077	0.165	0.188	0.163	0.092	0.168	0.171	0.177	0.207	0.04	0.025	0.028	0.086	0.033	0.028	0.109	0.068	0.109	0.145	0.118	0.113
18	rs264588	A	0.175	0.325	0.329	0.313	0.438	0.406	0.237	0.303	0.234	0.16	0.141	0.115	0.186	0.2	0.167	0.092	0.168	0.171	0.199	0.207	0.044	0.025	0.042	0.086	0.033	0.033	0.127	0.073	0.109	0.157	0.162	0.137
19	rs264631	G	0.194	0.394	0.412	0.379	0.465	0.459	0.338	0.393	0.307	0.167	0.141	0.12	0.202	0.206	0.165	0.092	0.178	0.171	0.177	0.207	0.044	0.025	0.042	0.086	0.033	0.033	0.128	0.073	0.109	0.157	0.162	0.142
20	rs3812718	T	0.493	0.34	0.319	0.359	0.363	0.365	0.323	0.344	0.307	0.445	0.336	0.413	0.558	0.441	0.596	0.481	0.514	0.619	0.677	0.702	0.541	0.53	0.533	0.571	0.61	0.472	0.581	0.583	0.583	0.599	0.588	0.554
21	rs2952768	C	0.386	0.293	0.241	0.374	0.266	0.341	0.227	0.303	0.323	0.409	0.43	0.351	0.346	0.535	0.404	0.471	0.322	0.433	0.452	0.343	0.354	0.374	0.313	0.353	0.396	0.341	0.511	0.476	0.484	0.506	0.52	0.569
22	rs4673993	C	0.285	0.095	0.083	0.056	0.075	0.106	0.111	0.147	0.115	0.307	0.328	0.308	0.261	0.341	0.294	0.228	0.178	0.243	0.473	0.369	0.313	0.293	0.346	0.293	0.297	0.332	0.491	0.485	0.448	0.483	0.475	0.559
23	rs2011425	G	0.138	0.077	0.083	0.081	0.062	0.035	0.101	0.082	0.094	0.101	0.125	0.077	0.08	0.135	0.208	0.155	0.135	0.243	0.269	0.247	0.094	0.086	0.159	0.061	0.071	0.089	0.221	0.204	0.208	0.227	0.23	0.235
24	rs887829	T	0.354	0.493	0.523	0.53	0.491	0.488	0.46	0.459	0.484	0.379	0.367	0.361	0.34	0.453	0.13	0.112	0.178	0.114	0.156	0.091	0.298	0.318	0.271	0.359	0.264	0.28	0.437	0.442	0.448	0.419	0.466	0.407
25	rs2228001	G	0.315	0.249	0.269	0.273	0.243	0.212	0.227	0.336	0.208	0.282	0.258	0.298	0.293	0.271	0.333	0.384	0.346	0.319	0.296	0.318	0.405	0.409	0.458	0.353	0.385	0.411	0.318	0.34	0.411	0.273	0.324	0.24
26	rs1801019	C	0.186	0.152	0.162	0.131	0.159	0.141	0.136	0.164	0.172	0.262	0.313	0.202	0.218	0.347	0.168	0.146	0.24	0.162	0.156	0.131	0.149	0.157	0.112	0.146	0.143	0.187	0.234	0.252	0.24	0.174	0.245	0.25
27	rs4961	T	0.208	0.049	0.032	0.056	0.08	0.029	0.056	0.057	0.031	0.174	0.227	0.139	0.165	0.188	0.452	0.544	0.563	0.457	0.371	0.313	0.205	0.217	0.187	0.222	0.258	0.149	0.2	0.175	0.156	0.221	0.201	0.25
28	rs145489027	A	0.301	0.098	0.083	0.091	0.093	0.088	0.096	0.18	0.083	0.228	0.258	0.188	0.186	0.3	0.456	0.442	0.394	0.405	0.548	0.505	0.333	0.364	0.29	0.424	0.313	0.28	0.437	0.427	0.448	0.419	0.417	0.471
29	rs1902023	A	0.454	0.398	0.431	0.308	0.447	0.382	0.419	0.361	0.411	0.395	0.273	0.471	0.473	0.306	0.423	0.422	0.433	0.424	0.441	0.394	0.513	0.55	0.505	0.515	0.544	0.486	0.541	0.553	0.495	0.448	0.637	0.554
30	rs61750900	T	0.044	0.036	0.014	0.04	0.035	0.024	0.03	0.041	0.068	0.072	0.047	0.115	0.074	0.035	0.001	0	0	0	0.005	0	0.091	0.096	0.112	0.061	0.126	0.065	0.031	0.034	0.036	0.029	0.029	0.025
31	rs4693075	G	0.34	0.449	0.495	0.47	0.389	0.412	0.47	0.525	0.411	0.425	0.32	0.462	0.356	0.535	0.129	0.117	0.154	0.124	0.14	0.111	0.354	0.353	0.402	0.258	0.429	0.332	0.334	0.364	0.375	0.326	0.279	0.328
32	rs2231142	T	0.119	0.013	0	0	0.013	0.018	0	0.074	0.01	0.141	0.203	0.106	0.133	0.147	0.291	0.311	0.322	0.257	0.215	0.343	0.094	0.116	0.061	0.091	0.143	0.07	0.097	0.068	0.104	0.122	0.088	0.108
33	rs4444903	A	0.395	0.238	0.25	0.253	0.195	0.2	0.247	0.311	0.24	0.419	0.383	0.476	0.489	0.3	0.306	0.296	0.293	0.286	0.36	0.303	0.609	0.601	0.617	0.626	0.593	0.608	0.459	0.505	0.516	0.36	0.485	0.417
34	rs1801394	G	0.364	0.246	0.259	0.303	0.208	0.129	0.242	0.32	0.276	0.281	0.281	0.413	0.277	0.123	0.263	0.252	0.303	0.252	0.253	0.253	0.523	0.561	0.393	0.616	0.566	0.495	0.524	0.481	0.51	0.535	0.51	0.588
35	rs6295	C	0.453	0.57	0.588	0.611	0.562	0.547	0.535	0.566	0.573	0.496	0.578	0.476	0.521	0.429	0.203	0.306	0.221	0.129	0.194	0.167	0.542	0.505	0.589	0.606	0.472	0.528	0.432	0.403	0.464	0.43	0.451	0.417
36	rs17244841	T	0.037	0.107	0.093	0.086	0.159	0.076	0.121	0.098	0.104	0.026	0.039	0.014	0.048	0.006	0.006	0	0.019	0.009	0	0	0.018	0.015	0.033	0.005	0.028	0.009	0.001	0	0	0	0.005	0
37	rs1042713	A	0.476	0.52	0.532	0.49	0.531	0.523	0.515	0.549	0.51	0.457	0.477	0.409	0.516	0.435	0.549	0.548	0.442	0.61	0.581	0.566	0.386	0.348	0.369	0.414	0.418	0.383	0.446	0.432	0.427	0.43	0.461	0.475
38	rs1800888	T	0.004	0	0	0	0	0	0	0	0	0	0	0	0	0	0	0	0	0	0	0	0.018	0.015	0.014	0.01	0.022	0.028	0.002	0	0.005	0	0.005	0
39	rs730012	C	0.17	0.041	0.009	0.051	0.066	0.024	0.02	0.049	0.068	0.222	0.234	0.216	0.245	0.194	0.151	0.165	0.159	0.09	0.161	0.182	0.312	0.323	0.285	0.394	0.324	0.243	0.182	0.189	0.151	0.169	0.201	0.196
40	rs1142345	C	0.039	0.067	0.06	0.116	0.04	0.076	0.04	0.098	0.052	0.058	0.047	0.909, 0.	0.021	0.065	0.022	0.005	0.019	0.029	0.032	0.025	0.029	0.03	0.014	0.025	0.033	0.042	0.017	0.024	0.01	0.029	0.005	0.02
41	rs56161402	T	0.006	0.02	0.023	0.01	0.026	0	0.045	0.016	0.01	0.003	0	0	0.005	0.006	0	0	0	0	0	0	0	0	0	0	0	0	0	0	0	0	0	0
42	rs1800460	T	0.013	0.003	0	0	0	0	0	0.025	0.005	0.04	0.039	0.048	0.011	0.065	0	0	0	0	0	0	0.028	0.03	0.014	0.025	0.028	0.042	0.004	0	0	0.017	0.005	0
43	rs56019966	T	0.001	0	0	0	0	0	0	0	0	0	0	0	0	0	0	0	0	0	0	0	0.003	0	0.005	0	0.005	0.005	0	0	0	0	0	0
44	rs1800462	G	0.002	0.001	0	0	0	0	0	0.008	0	0.006	0	0.005	0.011	0.006	0	0	0	0	0	0	0.006	0	0.009	0	0.011	0.009	0	0	0	0	0	0
45	rs746647	G	0.22	0.222	0.208	0.263	0.243	0.176	0.237	0.213	0.198	0.275	0.328	0.264	0.287	0.235	0.072	0.141	0.038	0.071	0.016	0.091	0.315	0.303	0.355	0.343	0.308	0.266	0.235	0.349	0.198	0.145	0.176	0.289
46	rs1800629	A	0.09	0.119	0.102	0.086	0.142	0.159	0.126	0.074	0.135	0.069	0.055	0.086	0.069	0.059	0.059	0.092	0.019	0.057	0.07	0.056	0.134	0.187	0.093	0.126	0.121	0.145	0.053	0.049	0.057	0.023	0.093	0.039
47	rs1360780	T	0.327	0.421	0.417	0.359	0.42	0.418	0.455	0.426	0.458	0.288	0.219	0.327	0.314	0.265	0.246	0.214	0.216	0.229	0.312	0.268	0.306	0.278	0.374	0.182	0.324	0.364	0.333	0.384	0.323	0.372	0.299	0.294
48	rs4713916	A	0.222	0.058	0.032	0.076	0.075	0.035	0.02	0.107	0.073	0.261	0.234	0.288	0.271	0.235	0.206	0.194	0.188	0.186	0.258	0.212	0.317	0.283	0.374	0.258	0.302	0.36	0.335	0.384	0.318	0.349	0.319	0.309
49	rs20455	A	0.46	0.146	0.125	0.207	0.128	0.118	0.101	0.213	0.156	0.666	0.68	0.591	0.622	0.794	0.476	0.524	0.51	0.476	0.478	0.389	0.637	0.631	0.654	0.657	0.659	0.589	0.541	0.544	0.552	0.564	0.524	0.524
50	rs1799971	G	0.223	0.009	0	0.005	0	0	0	0.049	0.026	0.2	0.227	0.221	0.16	0.2	0.393	0.349	0.486	0.295	0.446	0.394	0.162	0.151	0.178	0.182	0.121	0.173	0.418	0.398	0.37	0.442	0.431	0.451
51	rs4880	G	0.411	0.424	0.403	0.455	0.434	0.435	0.394	0.361	0.464	0.584	0.648	0.543	0.495	0.682	0.125	0.117	0.101	0.11	0.15	0.151	0.466	0.429	0.472	0.475	0.516	0.444	0.508	0.505	0.495	0.477	0.578	0.48
52	rs10455872	G	0.022	0.002	0	0	0.004	0	0	0.016	0	0.033	0.008	0.043	0.059	0.012	0.005	0	0	0	0.005	0.02	0.074	0.106	0.056	0.04	0.077	0.089	0.006	0	0.005	0.023	0	0.005
53	rs37973	G	0.396	0.195	0.162	0.111	0.234	0.235	0.121	0.311	0.24	0.435	0.453	0.346	0.457	0.506	0.48	0.456	0.438	0.51	0.478	0.52	0.44	0.475	0.393	0.444	0.472	0.425	0.507	0.534	0.484	0.593	0.417	0.52
54	rs7793837	T	0.415	0.86	0.912	0.843	0.872	0.859	0.914	0.762	0.813	0.336	0.367	0.37	0.308	0.3	0.178	0.155	0.197	0.133	0.14	0.263	0.258	0.202	0.238	0.258	0.242	0.341	0.278	0.248	0.25	0.302	0.328	0.265
55	rs52815063	T	0.005	0.002	0	0	0	0	0	0	0.01	0.003	0	0.01	0	0	0	0	0	0	0	0	0.017	0.015	0.005	0.02	0.022	0.023	0.002	0	0.01	0	0	0
56	rs7779029	C	0.168	0.365	0.366	0.369	0.42	0.365	0.338	0.295	0.365	0.082	0.047	0.154	0.085	0.018	0.152	0.121	0.168	0.167	0.194	0.111	0.047	0.066	0.065	0.02	0.022	0.056	0.106	0.063	0.094	0.105	0.172	0.098
57	rs1045642	A	0.395	0.15	0.125	0.141	0.19	0.147	0.116	0.189	0.151	0.428	0.477	0.428	0.442	0.377	0.398	0.379	0.481	0.305	0.425	0.404	0.518	0.566	0.467	0.576	0.527	0.463	0.575	0.568	0.51	0.611	0.593	0.593
58	rs776746	T	0.379	0.82	0.833	0.879	0.774	0.882	0.894	0.689	0.75	0.203	0.234	0.264	0.186	0.123	0.287	0.311	0.255	0.271	0.312	0.288	0.057	0.04	0.051	0.061	0.055	0.075	0.332	0.277	0.365	0.366	0.338	0.324

59	rs2740574	C	0.231	0.765	0.764	0.833	0.792	0.835	0.768	0.672	0.661	0.105	0.07	0.188	0.101	0.035	0.004	0	0	0	0	0.02	0.028	0.015	0.028	0.035	0.033	0.028	0.04	0.078	0.026	0.017	0.029	0.044
60	rs339097	G	0.043	0.144	0.185	0.182	0.102	0.1	0.121	0.147	0.167	0.011	0	0.024	0.016	0	0.014	0.01	0.005	0.029	0.016	0.01	0	0	0	0	0	0	0.006	0	0.005	0.012	0.01	0.005
61	rs10246939	T	0.479	0.523	0.537	0.505	0.58	0.512	0.53	0.41	0.536	0.313	0.328	0.365	0.404	0.135	0.323	0.32	0.433	0.319	0.285	0.253	0.539	0.566	0.444	0.621	0.577	0.5	0.638	0.578	0.589	0.669	0.701	0.657
62	rs1726866	A	0.425	0.333	0.31	0.298	0.398	0.382	0.313	0.287	0.323	0.285	0.313	0.322	0.378	0.118	0.324	0.325	0.433	0.319	0.285	0.253	0.539	0.571	0.444	0.621	0.577	0.495	0.638	0.578	0.589	0.669	0.701	0.657
63	rs713598	C	0.495	0.525	0.537	0.5	0.584	0.512	0.52	0.443	0.536	0.342	0.328	0.428	0.436	0.141	0.324	0.325	0.433	0.319	0.285	0.253	0.578	0.616	0.495	0.626	0.61	0.551	0.655	0.597	0.62	0.68	0.701	0.681
64	rs6977820	T	0.423	0.825	0.875	0.833	0.836	0.847	0.899	0.68	0.74	0.252	0.281	0.327	0.261	0.129	0.238	0.267	0.322	0.214	0.204	0.177	0.307	0.293	0.374	0.192	0.302	0.364	0.31	0.306	0.323	0.32	0.343	0.26
65	rs1801279	A	0.028	0.103	0.116	0.091	0.142	0.071	0.126	0.074	0.078	0.003	0	0.005	0.005	0	0	0	0	0	0	0	0.001	0	0.005	0	0	0	0	0	0	0	0	0
66	rs1041983	T	0.397	0.468	0.495	0.429	0.385	0.5	0.53	0.451	0.495	0.291	0.273	0.298	0.325	0.259	0.44	0.364	0.356	0.452	0.495	0.54	0.305	0.303	0.299	0.298	0.297	0.327	0.428	0.442	0.422	0.366	0.5	0.402
67	rs1801280	C	0.293	0.292	0.241	0.359	0.336	0.241	0.273	0.311	0.281	0.362	0.383	0.394	0.388	0.277	0.038	0.029	0.019	0.038	0.048	0.056	0.449	0.424	0.43	0.455	0.467	0.472	0.347	0.345	0.411	0.36	0.279	0.343
68	rs1799930	A	0.265	0.237	0.199	0.273	0.186	0.224	0.263	0.287	0.26	0.172	0.133	0.207	0.239	0.082	0.256	0.199	0.255	0.252	0.226	0.348	0.282	0.298	0.28	0.263	0.275	0.294	0.36	0.388	0.365	0.267	0.422	0.343
69	rs1208	G	0.323	0.395	0.37	0.46	0.438	0.347	0.389	0.361	0.375	0.373	0.445	0.389	0.394	0.277	0.04	0.029	0.019	0.043	0.048	0.061	0.438	0.399	0.435	0.429	0.451	0.477	0.363	0.345	0.422	0.39	0.299	0.368
70	rs1517114	C	0.317	0.424	0.495	0.399	0.416	0.388	0.404	0.418	0.432	0.339	0.32	0.409	0.346	0.259	0.122	0.068	0.13	0.114	0.172	0.131	0.363	0.369	0.388	0.318	0.385	0.355	0.309	0.311	0.307	0.308	0.299	0.319
71	rs6988229	T	0.238	0.597	0.62	0.571	0.624	0.588	0.586	0.574	0.599	0.184	0.18	0.173	0.192	0.194	0.005	0	0.01	0	0	0.015	0.193	0.253	0.229	0.131	0.225	0.131	0.079	0.058	0.094	0.093	0.059	0.093
72	rs7853758	A	0.203	0.34	0.366	0.303	0.358	0.377	0.364	0.238	0.333	0.233	0.18	0.192	0.218	0.341	0.156	0.126	0.111	0.143	0.21	0.197	0.137	0.187	0.131	0.061	0.137	0.168	0.112	0.121	0.083	0.116	0.128	0.108
73	rs885004	A	0.132	0.101	0.093	0.111	0.053	0.106	0.121	0.115	0.12	0.202	0.172	0.139	0.175	0.329	0.161	0.141	0.115	0.133	0.215	0.207	0.126	0.182	0.121	0.056	0.126	0.145	0.101	0.107	0.073	0.105	0.113	0.108
74	rs10306114	G	0.054	0.129	0.106	0.121	0.164	0.118	0.126	0.131	0.135	0.029	0.031	0.014	0.048	0.024	0	0	0	0	0	0	0.07	0.086	0.028	0.101	0.077	0.061	0.01	0.015	0.01	0.017	0	0.01
75	rs12777823	A	0.245	0.251	0.287	0.273	0.177	0.235	0.323	0.213	0.24	0.107	0.133	0.125	0.106	0.065	0.315	0.34	0.322	0.352	0.263	0.288	0.151	0.151	0.098	0.217	0.148	0.145	0.362	0.33	0.359	0.326	0.412	0.378
76	rs12248560	T	0.153	0.235	0.245	0.177	0.239	0.259	0.247	0.197	0.271	0.12	0.117	0.178	0.128	0.041	0.015	0.024	0.005	0.009	0.016	0.02	0.224	0.222	0.224	0.217	0.242	0.215	0.136	0.136	0.146	0.111	0.142	0.142
77	rs28399504	G	0.001	0	0	0	0	0	0	0	0	0.003	0.008	0.005	0	0	0.001	0.005	0	0	0	0	0.001	0	0	0	0	0.005	0	0	0	0	0	0
78	rs4986893	A	0.014	0.002	0	0.01	0	0	0	0	0.005	0	0	0	0	0	0.056	0.044	0.072	0.048	0.075	0.04	0	0	0	0	0	0	0.012	0.005	0.016	0.023	0.015	0.005
79	rs4244285	A	0.221	0.17	0.167	0.212	0.133	0.176	0.207	0.139	0.151	0.105	0.125	0.13	0.106	0.059	0.313	0.335	0.322	0.352	0.263	0.283	0.145	0.131	0.093	0.217	0.143	0.145	0.358	0.33	0.344	0.326	0.412	0.372
80	rs1799853	T	0.048	0.008	0	0	0.004	0	0	0.041	0.026	0.099	0.102	0.139	0.122	0.024	0.001	0	0	0.005	0	0	0.124	0.151	0.154	0.081	0.088	0.14	0.035	0.049	0.052	0.017	0.029	0.025
81	rs7900194	A	0.015	0.053	0.051	0.071	0.018	0.035	0.076	0.033	0.083	0.001	0	0.005	0	0	0	0	0	0	0	0	0.002	0.005	0	0	0	0.005	0.001	0	0.005	0	0	0
82	rs75838422	A	0.015	0.053	0.051	0.071	0.018	0.035	0.076	0.033	0.083	0.001	0	0.005	0	0	0	0	0	0	0	0	0.002	0.005	0	0	0	0.005	0.001	0	0.005	0	0	0
83	rs4917639	C	0.164	0.209	0.194	0.227	0.195	0.2	0.212	0.221	0.219	0.151	0.133	0.211	0.202	0.035	0.088	0.078	0.086	0.095	0.107	0.076	0.198	0.217	0.238	0.136	0.159	0.229	0.155	0.184	0.167	0.151	0.142	0.132
84	rs1057910	C	0.049	0.002	0	0	0	0	0	0.016	0.005	0.037	0.023	0.043	0.064	0.012	0.034	0.039	0.019	0.048	0.027	0.035	0.073	0.066	0.084	0.056	0.071	0.084	0.109	0.131	0.099	0.116	0.098	0.103
85	rs28371686	G	0.005	0.017	0.028	0.01	0.009	0.006	0.025	0.025	0.016	0.001	0	0.005	0	0	0	0	0	0	0	0	0	0	0	0	0	0	0	0	0	0	0	0
86	rs10509681	C	0.046	0.008	0	0	0.004	0	0	0.033	0.031	0.099	0.102	0.144	0.117	0.024	0.001	0	0	0.005	0	0	0.118	0.131	0.131	0.081	0.093	0.149	0.03	0.039	0.047	0.017	0.02	0.025
87	rs11598702	C	0.256	0.185	0.185	0.202	0.142	0.212	0.167	0.189	0.208	0.307	0.281	0.308	0.362	0.265	0.234	0.252	0.197	0.191	0.263	0.273	0.353	0.384	0.322	0.247	0.467	0.355	0.237	0.204	0.266	0.209	0.294	0.211
88	rs7903146	T	0.228	0.26	0.241	0.278	0.23	0.229	0.247	0.361	0.276	0.235	0.219	0.322	0.239	0.135	0.023	0.024	0.029	0.029	0.021	0.01	0.317	0.313	0.374	0.227	0.258	0.397	0.299	0.282	0.25	0.279	0.338	0.338
89	rs1801253	G	0.298	0.43	0.477	0.308	0.456	0.447	0.47	0.369	0.453	0.195	0.172	0.231	0.25	0.106	0.212	0.214	0.183	0.238	0.204	0.222	0.315	0.328	0.346	0.338	0.297	0.266	0.266	0.228	0.276	0.308	0.27	0.255
90	rs5219	T	0.263	0.023	0	0.005	0.009	0	0	0.139	0.057	0.292	0.406	0.288	0.197	0.318	0.338	0.379	0.332	0.395	0.226	0.348	0.353	0.384	0.285	0.444	0.264	0.383	0.396	0.417	0.443	0.413	0.304	0.407
91	rs1695	G	0.353	0.48	0.398	0.51	0.535	0.471	0.54	0.459	0.438	0.475	0.563	0.37	0.356	0.671	0.179	0.184	0.101	0.191	0.22	0.202	0.331	0.394	0.294	0.283	0.319	0.364	0.294	0.311	0.292	0.221	0.314	0.324
92	rs716274	G	0.437	0.432	0.389	0.379	0.5	0.453	0.419	0.541	0.38	0.405	0.328	0.51	0.463	0.271	0.288	0.199	0.288	0.271	0.366	0.323	0.593	0.581	0.626	0.591	0.566	0.598	0.46	0.461	0.505	0.355	0.5	0.466
93	rs11212617	A	0.469	0.209	0.181	0.187	0.177	0.241	0.177	0.369	0.208	0.64	0.594	0.606	0.66	0.694	0.388	0.359	0.404	0.386	0.393	0.399	0.617	0.55	0.692	0.581	0.593	0.659	0.627	0.636	0.703	0.611	0.564	0.623
94	rs1076560	A	0.23	0.083	0.093	0.096	0.044	0.082	0.091	0.115	0.078	0.27	0.383	0.192	0.138	0.423	0.416	0.452	0.394	0.362	0.457	0.419	0.15	0.141	0.154	0.167	0.165	0.126	0.289	0.277	0.266	0.279	0.289	0.333
95	rs1799978	C	0.119	0.173	0.13	0.197	0.279	0.206	0.111	0.139	0.13	0.076	0.117	0.024	0.059	0.129	0.178	0.199	0.101	0.176	0.21	0.207	0.06	0.045	0.037	0.091	0.071	0.056	0.077	0.058	0.115	0.041	0.064	0.103
96	rs1954787	T	0.498	0.896	0.926	0.859	0.951	0.935	0.904	0.828	0.833	0.367	0.336	0.462	0.383	0.259	0.169	0.243	0.063	0.181	0.161	0.197	0.474	0.465	0.481	0.515	0.451	0.458	0.416	0.495	0.396	0.343	0.412	0.422
97	rs5443	T	0.492	0.819	0.852	0.752	0.819	0.888	0.808	0.779	0.828	0.379	0.375	0.356	0.489	0.288	0.503	0.461	0.438	0.5	0.581	0.545	0.307	0.364	0.304	0.222	0.346	0.304	0.31	0.325	0.344	0.355	0.25	0.284
98	rs4149015	A	0.055	0.007	0	0.03	0	0.006	0.005	0.008	0	0.037	0.023	0.043	0.069	0.006	0.131	0.121	0.115	0.119	0.145	0.157	0.065	0.04	0.079	0.096	0.022	0.079	0.043	0.058	0.021	0.041	0.044	0.049
99	rs4149056	C	0.088	0.014	0.009	0.02	0	0	0	0.066	0.021	0.134	0.078	0.12	0.181	0.141	0.123	0.136	0.12	0.119	0.14	0.101	0.161	0.146	0.215	0.182	0.143	0.117	0.043	0.019	0.036	0.052	0.044	0.064
100	rs11045879	C	0.219	0.189	0.157	0.202	0.243	0.212	0.141	0.18	0.182	0.173	0.109	0.178	0.197	0.188	0.453	0.456	0.385	0.452	0.538	0.444	0.19	0.157	0.224	0.293	0.148	0.126	0.082	0.058	0.078	0.145	0.049	0.088
101	rs2069707	C	0.02	0.008	0.005	0.02	0.009	0	0	0.008	0.016	0.04	0.031	0.053	0.064	0.006	0.001	0	0	0	0	0.005	0.056	0.071	0.061	0.03	0.06	0.056	0.002	0.005	0	0	0	0.005
102	rs7297610	T	0.116	0.328	0.37	0.313	0.354	0.377	0.293	0.23	0.323	0.072	0.055	0.086	0.09	0.047	0.001	0.005	0	0	0	0	0.074	0.081	0.112	0.025	0.06	0.084	0.021	0.029	0.021	0.023	0.025	0.01
103	rs7997012	A	0.273	0.014	0	0	0.004	0.006	0	0.066	0.047	0.352	0.32	0.303	0.34	0.447	0.258	0.257	0.173	0.295	0.333	0.237	0.429	0.419	0.379	0.53	0.44	0.388	0.42	0.524	0.417	0.355	0.378	0.417
104	rs554405994	GGAGTC	0.016	0.002	0	0.005	0.004	0	0	0	0	0.039	0.031	0.005	0.021	0.106	0.048	0.053	0.034	0.067	0.038	0.045	0.003	0.005	0	0.01	0	0	0.002	0	0	0.006	0.005	0
105	rs186364861	A	0.003	0	0	0	0	0	0	0	0	0	0	0	0	0	0.014	0.015	0.014	0.029	0.011	0	0	0	0	0	0	0	0.001	0	0	0.006	0	0
106	rs116855232	T	0.04	0.001	0	0	0	0	0	0.008	0	0.045	0.047	0.005	0.021	0.118	0.095	0.131	0.067	0.162	0.048	0.061	0.002	0	0	0.01	0	0	0.07	0.039	0.083	0.064	0.083	0.078
107	rs147390019	A	0.001	0	0	0	0	0	0	0	0	0.007	0.016	0	0.016	0	0.001	0	0.005	0	0	0	0	0	0	0	0	0	0	0	0	0	0	0
108	rs1751034	C	0.206	0.266	0.273	0.328	0.226	0.218	0.273	0.205	0.318	0.246	0.266	0.216	0.202	0.318	0.215	0.194	0.332	0.181	0.194	0.172	0.189	0.141	0.234	0.167	0.165	0.229	0.102	0.146	0.109	0.093	0.073	0.088
109	rs1719247	C	0.412	0.219	0.176	0.202	0.248	0.206	0.167	0.32	0.25	0.444	0.383	0.601	0.532	0.2	0.194	0.146	0.115	0.181	0.22	0.313	0.728	0.798	0.706	0.667	0.736	0.734	0.554	0.568	0.609	0.506	0.51	0.574
110	rs1346268	C	0.451	0.319	0.255	0.338	0.416	0.394	0.258	0.213	0.323	0.522	0.609	0.341	0.42	0.788	0.806	0.85	0.88	0.819	0.796	0.682	0.275	0.197	0.308	0.333	0.28	0.257	0.393	0.374	0.333	0.448	0.417	0.397
111	rs578776	G	0.445	0.396	0.352	0.323	0.411	0.429	0.409	0.377	0.474	0.404	0.398	0.495	0.527	0.159	0.199	0.194	0.168	0.209	0.134	0.288	0.719	0.748	0.692	0.702	0.714	0.738	0.513	0.515	0.526	0.407	0.574	0.529
112	rs1051730	A	0.168	0.087	0.093	0.045	0.075	0.035	0.091	0.147	0.141	0.216	0.188	0.231	0.356	0.065	0.027	0.029	0.019	0.043	0.021	0.02	0.369	0.384	0.383	0.303	0.28	0.477	0.183	0.204	0.208	0.145	0.181	0.172
113	rs7294	T	0.42	0.454	0.514	0.429	0.451	0.359	0.48	0.475	0.458	0.402	0.352	0.341	0.356	0.565	0.112	0.044	0.096	0.11	0.167	0.151	0.366	0.313	0.336	0.414	0.418	0.418	0.759	0.665	0.693	0.785	0.824	0.828
114	rs2359612	A	0.39	0.178	0.176	0.182	0.168	0.188	0.177	0.221	0.156	0.425	0.469	0.418	0.436	0.388	0.885	0.956	0.904	0.89	0.823	0.843	0.388	0.429	0.477	0.308	0.357	0.357	0.145	0.175	0.198	0.157	0.108	0.093
115	rs8050894	G	0.416	0.256	0.227	0.167	0.283	0.341	0.212	0.295	0.297	0.442	0.508	0.447	0.442	0.388	0.885	0.956	0.904	0.89	0.823	0.843	0.4	0.434	0.5	0.308	0.368	0.368	0.148	0.18	0.198	0.157	0.113	0.098
116	rs9934438	A	0.356	0.054	0.028	0.035	0.066	0.053	0.025	0.147	0.063	0.412	0.469	0.389	0.42	0.388	0.885	0.956	0.904	0.89	0.823	0.843	0.388	0.429	0.477	0.308	0.357	0.357	0.145	0.175	0.198	0.157	0.108	0.093
117	rs17708472	A	0.094	0.033	0.018	0.061	0.022	0.006	0.005	0.074	0.063	0.14	0.148	0.178	0.181	0.041	0.003	0	0	0	0.011	0.005	0.234	0.253	0.164	0.278	0.214	0.214	0.092	0.155	0.109	0.058	0.059	0.073
118	rs2884737	C	0.091	0.009	0	0.005	0.004	0	0	0.066	0.01	0.176	0.141	0.303	0.186	0.035	0.001	0.005	0	0	0	0	0.256	0.268	0.327	0.187	0.214	0.214	0.068	0.068	0.125	0.058	0.034	0.054
119	rs9923231	T	0.356	0.054	0.028	0.035	0.066	0.053	0.025	0.147	0.063	0.411	0.469	0.389	0.42	0.382	0.885	0.956	0.904	0.89	0.823	0.843	0.388	0.429	0.477	0.308	0.357	0.357	0.145	0.175	0.198	0.157	0.108	0.093
120	rs7196161	G	0.474	0.487	0.449	0.439	0.487	0.606	0.505	0.459	0.474	0.429	0.484	0.433	0.425	0.388	0.885	0.956	0.904	0.89	0.823	0.843	0.393	0.439	0.467	0.328	0.346	0.346	0.15	0.175	0.208	0.151	0.118	0.103

121	rs1532624	A	0.313	0.104	0.06	0.066	0.124	0.088	0.111	0.156	0.146	0.344	0.398	0.327	0.308	0.365	0.291	0.325	0.284	0.329	0.258	0.253	0.426	0.455	0.416	0.424	0.429	0.429	0.48	0.544	0.458	0.424	0.436	0.524
122	rs2232228	G	0.342	0.059	0.065	0.025	0.044	0.035	0.035	0.131	0.104	0.434	0.5	0.385	0.367	0.482	0.515	0.544	0.462	0.5	0.565	0.51	0.438	0.379	0.472	0.394	0.494	0.494	0.383	0.354	0.328	0.483	0.372	0.392
123	rs6065	T	0.132	0.251	0.245	0.308	0.226	0.288	0.207	0.246	0.245	0.154	0.133	0.154	0.144	0.182	0.07	0.063	0.144	0.033	0.065	0.045	0.087	0.056	0.084	0.136	0.066	0.093	0.062	0.068	0.068	0.07	0.044	0.064
124	rs1042522	G	0.457	0.669	0.639	0.748	0.708	0.612	0.682	0.598	0.656	0.317	0.313	0.313	0.282	0.365	0.414	0.452	0.317	0.4	0.473	0.434	0.285	0.242	0.276	0.303	0.308	0.299	0.492	0.422	0.521	0.424	0.544	0.539
125	rs12943590	A	0.316	0.184	0.162	0.187	0.221	0.2	0.131	0.221	0.177	0.318	0.375	0.274	0.298	0.353	0.449	0.442	0.38	0.495	0.441	0.49	0.273	0.298	0.28	0.217	0.308	0.266	0.397	0.34	0.417	0.424	0.402	0.407
126	rs1876828	T	0.086	0.015	**0**	0.005	0.004	**0**	**0**	0.082	0.042	0.159	0.172	0.202	0.181	0.071	0.001	**0**	0.005	**0**	**0**	**0**	0.24	0.207	0.364	0.106	0.247	0.262	0.061	0.092	0.099	0.041	0.029	0.044
127	rs16960228	A	0.157	0.337	0.407	0.288	0.42	0.294	0.308	0.254	0.333	0.127	0.094	0.101	0.144	0.165	0.163	0.146	0.178	0.162	0.161	0.167	0.059	0.051	0.037	0.035	0.077	0.093	0.032	0.039	0.005	0.058	0.034	0.025
128	rs4149601	A	0.276	0.361	0.375	0.5	0.266	0.294	0.369	0.352	0.37	0.209	0.117	0.322	0.277	0.065	0.211	0.209	0.106	0.209	0.231	0.308	0.354	0.348	0.364	0.308	0.313	0.425	0.196	0.228	0.188	0.192	0.211	0.162
129	rs17782313	C	0.24	0.278	0.296	0.278	0.23	0.224	0.313	0.279	0.323	0.134	0.133	0.216	0.112	0.059	0.186	0.184	0.264	0.157	0.129	0.192	0.24	0.258	0.28	0.162	0.236	0.257	0.32	0.282	0.365	0.262	0.319	0.368
130	rs489693	A	0.351	0.504	0.532	0.485	0.531	0.577	0.439	0.443	0.5	0.262	0.305	0.385	0.181	0.171	0.216	0.209	0.298	0.205	0.15	0.212	0.322	0.353	0.346	0.222	0.341	0.346	0.377	0.364	0.448	0.302	0.343	0.422
131	rs2108622	T	0.237	0.083	0.056	0.111	0.066	0.106	0.045	0.09	0.115	0.238	0.25	0.288	0.282	0.118	0.214	0.218	0.231	0.2	0.199	0.222	0.29	0.247	0.336	0.217	0.286	0.355	0.413	0.437	0.391	0.413	0.422	0.402
132	rs11881222	G	0.251	0.307	0.278	0.328	0.345	0.282	0.288	0.385	0.266	0.372	0.445	0.322	0.383	0.365	0.084	0.068	0.101	0.052	0.107	0.096	0.293	0.268	0.369	0.247	0.28	0.294	0.219	0.214	0.234	0.163	0.23	0.245
133	rs12979860	T	0.356	0.669	0.676	0.515	0.73	0.653	0.712	0.68	0.714	0.399	0.461	0.365	0.415	0.377	0.08	0.063	0.101	0.048	0.102	0.091	0.309	0.273	0.397	0.268	0.308	0.294	0.233	0.238	0.24	0.198	0.25	0.235
134	rs8099917	G	0.131	0.042	0.032	0.076	0.035	0.012	0.02	0.098	0.042	0.277	0.352	0.192	0.271	0.329	0.076	0.063	0.096	0.052	0.097	0.076	0.168	0.151	0.206	0.131	0.17	0.178	0.167	0.16	0.188	0.111	0.191	0.176
135	rs111033610	G	0.002	**0**	**0**	**0**	**0**	**0**	**0**	**0**	**0**	**0**	**0**	**0**	**0**	**0**	0.008	0.01	**0**	0.009	0.005	0.015	**0**	**0**	**0**	**0**	**0**	**0**	**0**	**0**	**0**	**0**	**0**	**0**
136	rs1801272	T	0.009	0.001	**0**	**0**	**0**	**0**	**0**	0.008	**0**	0.007	0.016	0.005	**0**	0.012	**0**	**0**	**0**	**0**	**0**	**0**	0.034	0.035	0.047	0.02	0.028	0.037	0.006	0.01	**0**	0.012	0.01	**0**
137	rs186335453	T	**0**	0.002	**0**	0.01	**0**	**0**	**0**	**0**	**0**	**0**	**0**	**0**	**0**	**0**	**0**	**0**	**0**	**0**	**0**	**0**	**0**	**0**	**0**	**0**	**0**	**0**	**0**	**0**	**0**	**0**	**0**	**0**
138	rs139801276	C	0.002	0.008	0.005	0.015	0.009	0.018	0.005	0.008	**0**	**0**	**0**	**0**	**0**	**0**	**0**	**0**	**0**	**0**	**0**	**0**	**0**	**0**	**0**	**0**	**0**	**0**	**0**	**0**	**0**	**0**	**0**	**0**
139	rs4803419	T	0.289	0.083	0.037	0.086	0.08	0.076	0.04	0.156	0.135	0.352	0.422	0.274	0.33	0.418	0.435	0.495	0.418	0.476	0.371	0.409	0.32	0.283	0.271	0.374	0.302	0.369	0.342	0.359	0.318	0.36	0.333	0.338
140	rs3745274	T	0.316	0.374	0.403	0.359	0.35	0.353	0.414	0.352	0.38	0.373	0.313	0.346	0.367	0.459	0.215	0.16	0.221	0.157	0.328	0.222	0.236	0.278	0.304	0.157	0.231	0.206	0.381	0.403	0.38	0.366	0.397	0.358
141	rs36079186	C	0.001	0.002	**0**	0.015	**0**	**0**	**0**	**0**	**0**	**0**	**0**	**0**	**0**	**0**	**0**	**0**	**0**	**0**	**0**	**0**	**0**	**0**	**0**	**0**	**0**	**0**	0.001	**0**	0.005	**0**	**0**	**0**
142	rs25487	T	0.26	0.11	0.111	0.096	0.093	0.076	0.086	0.189	0.151	0.313	0.273	0.288	0.372	0.306	0.235	0.252	0.279	0.209	0.199	0.232	0.366	0.353	0.374	0.333	0.341	0.421	0.344	0.393	0.365	0.343	0.328	0.289
143	rs3212986	A	0.295	0.291	0.333	0.263	0.252	0.282	0.278	0.311	0.328	0.354	0.5	0.313	0.271	0.388	0.299	0.306	0.221	0.314	0.306	0.348	0.251	0.247	0.285	0.263	0.22	0.234	0.301	0.282	0.307	0.366	0.309	0.25
144	rs11615	A	0.331	0.037	0.018	0.035	0.031	0.024	0.005	0.098	0.073	0.388	0.258	0.495	0.479	0.253	0.262	0.252	0.293	0.267	0.226	0.268	0.622	0.636	0.542	0.631	0.681	0.631	0.46	0.519	0.505	0.355	0.451	0.456
145	rs1127354	A	0.089	0.045	0.046	0.081	0.044	0.029	0.061	0.025	0.016	0.042	0.047	0.077	0.027	0.012	0.169	0.083	0.13	0.209	0.237	0.192	0.071	0.076	0.107	0.051	0.055	0.061	0.122	0.136	0.12	0.099	0.113	0.137
146	rs7270101	C	0.059	0.071	0.106	0.126	0.044	0.024	0.071	0.008	0.088	0.082	0.07	0.115	0.08	0.053	**0**	**0**	**0**	**0**	**0**	**0**	0.129	0.146	0.103	0.056	0.192	0.154	0.015	0.029	0.021	0.012	0.01	0.005
147	rs1056892	A	0.427	0.51	0.509	0.455	0.526	0.518	0.566	0.443	0.526	0.261	0.281	0.274	0.293	0.194	0.405	0.388	0.337	0.452	0.382	0.465	0.354	0.323	0.416	0.333	0.374	0.322	0.532	0.544	0.474	0.593	0.529	0.524
148	rs13306278	T	0.077	0.008	**0**	0.015	**0**	**0**	**0**	0.016	0.026	0.088	0.086	0.106	0.117	0.035	0.016	0.019	0.019	0.005	0.011	0.025	0.151	0.197	0.14	0.091	0.181	0.149	0.151	0.18	0.12	0.145	0.162	0.147
149	rs4680	A	0.369	0.281	0.306	0.288	0.257	0.235	0.278	0.271	0.323	0.378	0.398	0.399	0.362	0.353	0.28	0.316	0.284	0.281	0.269	0.247	0.5	0.465	0.453	0.591	0.527	0.472	0.441	0.437	0.521	0.442	0.363	0.446
150	rs2298383	T	0.401	0.219	0.157	0.283	0.283	0.2	0.177	0.254	0.182	0.516	0.648	0.5	0.505	0.447	0.47	0.524	0.442	0.558	0.366	0.429	0.587	0.591	0.584	0.566	0.632	0.565	0.302	0.218	0.349	0.291	0.294	0.358
151	rs1135840	C	0.401	0.324	0.324	0.253	0.354	0.235	0.338	0.426	0.359	0.524	0.602	0.428	0.505	0.606	0.296	0.243	0.5	0.257	0.247	0.222	0.454	0.434	0.421	0.525	0.445	0.449	0.472	0.408	0.573	0.5	0.466	0.426
152	rs267608319	T	0.001	**0**	**0**	**0**	**0**	**0**	**0**	**0**	**0**	0.006	**0**	0.019	**0**	**0**	**0**	**0**	**0**	**0**	**0**	**0**	0.002	**0**	0.005	**0**	**0**	0.005	**0**	**0**	**0**	**0**	**0**	**0**
153	rs16947	A	0.359	0.554	0.56	0.646	0.535	0.612	0.566	0.418	0.495	0.327	0.258	0.389	0.308	0.324	0.14	0.165	0.139	0.133	0.129	0.131	0.343	0.323	0.374	0.323	0.308	0.379	0.362	0.442	0.318	0.25	0.378	0.402
154	rs35742686	-	0.006	0.002	**0**	**0**	**0**	**0**	**0**	0.016	0.005	0.006	**0**	0.005	0.016	**0**	**0**	**0**	**0**	**0**	**0**	**0**	0.019	0.02	**0**	0.035	0.033	0.009	0.002	0.005	**0**	0.006	**0**	**0**
155	rs3892097	T	0.093	0.06	0.056	0.025	0.04	0.041	0.076	0.123	0.088	0.13	0.125	0.154	0.165	0.065	0.002	0.005	**0**	**0**	**0**	0.005	0.186	0.227	0.187	0.136	0.242	0.145	0.109	0.126	0.078	0.122	0.108	0.113
156	rs5030865	T	0.002	**0**	**0**	**0**	**0**	**0**	**0**	**0**	**0**	**0**	**0**	**0**	**0**	**0**	0.01	0.005	0.005	**0**	0.016	0.025	**0**	**0**	**0**	**0**	**0**	**0**	**0**	**0**	**0**	**0**	**0**	**0**
157	rs5030655	-	0.005	0.001	**0**	**0**	**0**	**0**	**0**	0.008	**0**	0.003	**0**	**0**	0.011	**0**	**0**	**0**	**0**	**0**	**0**	**0**	0.02	0.02	0.019	0.045	0.011	0.005	0.001	**0**	0.005	**0**	**0**	**0**
158	rs267608275	-	0.031	0.117	0.116	0.187	0.155	0.1	0.086	0.049	0.094	0.003	**0**	0.005	0.005	**0**	**0**	**0**	**0**	**0**	**0**	**0**	**0**	**0**	**0**	**0**	**0**	**0**	**0**	**0**	**0**	**0**	**0**	**0**
159	rs1065852	A	0.238	0.113	0.106	0.035	0.115	0.165	0.091	0.156	0.146	0.148	0.148	0.178	0.186	0.071	0.571	0.602	0.361	0.614	0.629	0.662	0.202	0.242	0.206	0.146	0.247	0.173	0.165	0.15	0.104	0.256	0.152	0.172

* The values of the MAFs are represented with color codes (light blue indicates MAF = 0, light green indicates MAF from >0 to < 0.1, moderately darker green indicates MAF from ≥ 0.1 to < 0.2, dark green indicates MAF from ≥ 0.2 to < 0.3, dark blue indicates MAF from ≥ 0.3 to < 0.4 and red indicates MAF≥ 0.4). MAFs with ≥ are presented with bold fonts. List of these populations is given in supplementary table 1.
