# Supplementary material for: Heterogeneity in the distribution of 159 drug-response related SNPs in world populations and their genetic relatedness
Source: PLoS One. 2020 Jan 23;15(1):e0228000. doi: 10.1371/journal.pone.0228000 (PMC6977754; doi:10.1371/journal.pone.0228000)
Supplement: S2 Table — (DOC) [file pone.0228000.s003.doc]

**S2 table:** MAFs of *CYP* genes in the super-populations.

| **SNP** | **Gene** | **ALL** | **Super-population** | | | | |
| --- | --- | --- | --- | --- | --- | --- | --- |
|  |  |  | **AFR** | **AMR** | **EAS** | **EUR** | **SAS** |
| rs1135840 | *CYP2D6* | 0.401 | 0.324 | 0.524 | 0.296 | 0.454 | 0.472 |
| rs267608319 | *CYP2D6* | 0.001 | 0.000 | 0.006 | 0.000 | 0.002 | 0.000 |
| rs16947 | *CYP2D6* | 0.359 | 0.554 | 0.327 | 0.140 | 0.343 | 0.362 |
| rs3892097 | *CYP2D6* | 0.093 | 0.060 | 0.130 | 0.002 | 0.186 | 0.109 |
| rs5030865 | *CYP2D6* | 0.002 | 0.000 | 0.000 | 0.010 | 0.000 | 0.000 |
| rs1065852 | *CYP2D6* | 0.238 | 0.113 | 0.148 | 0.571 | 0.202 | 0.165 |
| rs12248560 | *CYP2C19* | 0.153 | 0.235 | 0.120 | 0.015 | 0.224 | 0.136 |
| rs28399504 | *CYP2C19* | 0.001 | 0.000 | 0.003 | 0.001 | 0.001 | 0.000 |
| rs4986893 | *CYP2C19* | 0.014 | 0.002 | 0.000 | 0.056 | 0.000 | 0.012 |
| rs4244285 | *CYP2C19* | 0.221 | 0.170 | 0.105 | 0.313 | 0.145 | 0.358 |
| rs1799853 | *CYP2C9* | 0.048 | 0.008 | 0.099 | 0.001 | 0.124 | 0.035 |
| rs7900194 | *CYP2C9* | 0.015 | 0.053 | 0.001 | 0.000 | 0.002 | 0.001 |
| rs4917639 | *CYP2C9* | 0.164 | 0.209 | 0.151 | 0.088 | 0.198 | 0.155 |
| rs1057910 | *CYP2C9* | 0.049 | 0.002 | 0.037 | 0.034 | 0.073 | 0.109 |
| rs28371686 | *CYP2C9* | 0.005 | 0.017 | 0.001 | 0.000 | 0.000 | 0.000 |
| rs10509681 | *CYP2C8* | 0.046 | 0.008 | 0.099 | 0.001 | 0.118 | 0.030 |
| rs111033610 | *CYP2A6* | 0.002 | 0.000 | 0.000 | 0.008 | 0.000 | 0.000 |
| rs1801272 | *CYP2A6* | 0.009 | 0.001 | 0.007 | 0.000 | 0.034 | 0.006 |
| rs186335453 | *CYP2B6* | 0.000 | 0.002 | 0.000 | 0.000 | 0.000 | 0.000 |
| rs139801276 | *CYP2B6* | 0.002 | 0.008 | 0.000 | 0.000 | 0.000 | 0.000 |
| rs4803419 | *CYP2B6* | 0.289 | 0.083 | 0.352 | 0.435 | 0.320 | 0.342 |
| rs3745274 | *CYP2B6* | 0.316 | 0.374 | 0.373 | 0.215 | 0.236 | 0.381 |
| rs36079186 | *CYP2B6* | 0.001 | 0.002 | 0.000 | 0.000 | 0.000 | 0.001 |
| rs25487 | *CYP2B6* | 0.260 | 0.110 | 0.313 | 0.235 | 0.366 | 0.344 |
| rs2108622 | *CYP2F2* | 0.237 | 0.083 | 0.238 | 0.214 | 0.290 | 0.413 |
| rs776746 | *CYP3A5* | 0.379 | 0.820 | 0.203 | 0.287 | 0.057 | 0.332 |
| rs2740574 | *CYP3A4* | 0.231 | 0.765 | 0.105 | 0.004 | 0.028 | 0.040 |
| rs1056836 | *CYP2B1* | 0.385 | 0.817 | 0.277 | 0.091 | 0.398 | 0.169 |
